# *De Novo* Prediction of PTBP1 Binding and Splicing Targets Reveals Unexpected Features of Its RNA Recognition and Function

**DOI:** 10.1371/journal.pcbi.1003442

**Published:** 2014-01-30

**Authors:** Areum Han, Peter Stoilov, Anthony J. Linares, Yu Zhou, Xiang-Dong Fu, Douglas L. Black

**Affiliations:** 1 Department of Bioengineering, University of California at Los Angeles, Los Angeles, California, United States of America; 2 Department of Biochemistry, School of Medicine, West Virginia University, Morgantown, West Virginia, United States of America; 3 Molecular Biology Interdepartmental Graduate Program, University of California at Los Angeles, Los Angeles, California, United States of America; 4 Medical Scientist Training Program, University of California at Los Angeles, Los Angeles, California, United States of America; 5 Department of Cellular and Molecular Medicine, University of California at San Diego, La Jolla, California, United States of America; 6 Department of Microbiology, Immunology and Molecular Genetics, University of California at Los Angeles, Los Angeles, California, United States of America; 7 Howard Hughes Medical Institute, University of California at Los Angeles, Los Angeles, California, United States of America; Center for Genomic Regulation, Spain

## Abstract

The splicing regulator Polypyrimidine Tract Binding Protein (PTBP1) has four RNA binding domains that each binds a short pyrimidine element, allowing recognition of diverse pyrimidine-rich sequences. This variation makes it difficult to evaluate PTBP1 binding to particular sites based on sequence alone and thus to identify target RNAs. Conversely, transcriptome-wide binding assays such as CLIP identify many *in vivo* targets, but do not provide a quantitative assessment of binding and are informative only for the cells where the analysis is performed. A general method of predicting PTBP1 binding and possible targets in any cell type is needed. We developed computational models that predict the binding and splicing targets of PTBP1. A Hidden Markov Model (HMM), trained on CLIP-seq data, was used to score probable PTBP1 binding sites. Scores from this model are highly correlated (ρ = −0.9) with experimentally determined dissociation constants. Notably, we find that the protein is not strictly pyrimidine specific, as interspersed Guanosine residues are well tolerated within PTBP1 binding sites. This model identifies many previously unrecognized PTBP1 binding sites, and can score PTBP1 binding across the transcriptome in the absence of CLIP data. Using this model to examine the placement of PTBP1 binding sites in controlling splicing, we trained a multinomial logistic model on sets of PTBP1 regulated and unregulated exons. Applying this model to rank exons across the mouse transcriptome identifies known PTBP1 targets and many new exons that were confirmed as PTBP1-repressed by RT-PCR and RNA-seq after PTBP1 depletion. We find that PTBP1 dependent exons are diverse in structure and do not all fit previous descriptions of the placement of PTBP1 binding sites. Our study uncovers new features of RNA recognition and splicing regulation by PTBP1. This approach can be applied to other multi-RRM domain proteins to assess binding site degeneracy and multifactorial splicing regulation.

## Author Summary

A key step in the regulation of mammalian genes is the splicing of the messenger RNA precursor to produce a mature mRNA that can be translated into a particular protein needed by the cell. Through the process of alternative splicing, mRNAs encoding different proteins can be derived from the same primary gene transcript. The regulation of this process plays essential roles in the development of differentiated tissues and is mediated by special pre-mRNA binding proteins. To understand how these proteins control gene expression, one must characterize what they recognize in RNA and identify these binding sites across the genome in order to predict their targets. Models that allow this prediction are essential to understanding developmental regulatory programs and their perturbation by disease causing mutations. In this study, we use statistical methods to build models of RNA recognition by the important splicing regulator PTBP1 and then apply these models to predict PTBP1 regulation of new gene transcripts. We show that PTBP1 has different specificity for RNA than was previously recognized and that its target exons are more diverse than was known before. There are many similar splicing regulators in mammalian cells, and these analyses provide a general framework for the computational analysis of their RNA binding and target identification.

## Introduction

Alternative splicing of pre-mRNA commonly determines the protein output of mammalian genes, with most genes generating multiple mRNA and protein products [1]. A typical alternative exon is affected by multiple pre-mRNA binding proteins that may either enhance or repress splicing [2]. The expression and activity of these splicing regulatory proteins can vary with development, cell type, or cellular stimulus [3]. This complex combinatorial regulation can be seen in the conserved sequences within and surrounding alternative exons, which generally contain the binding sites for many different regulators. These sequences make up what is sometimes called the splicing code as they determine where and when the exon is spliced into an mRNA [4], [5], [6], [7]. Such a code should allow the development of models that predict exon regulation based solely on the RNA binding affinity of the many regulatory proteins and their other interactions. However, this is not currently feasible, in part due to our incomplete understanding of RNA recognition by the splicing regulators and their mechanisms of action.

Whole-transcriptome crosslinking methods for individual proteins *in vivo* are allowing the identification of large numbers of protein/RNA interaction sites [8], [9], [10], [11]. These data can be overlapped with functional data on splicing to identify possible direct target exons for particular proteins [12], [13], [14], [15]. However, there are limitations in the interpretation of these data. Crosslinking efficiency can vary between different proteins and between individual binding sites, making it difficult to relate the crosslinking signal to the actual binding affinity. These signals are also dependent on the expression of the bound RNA, and since these data are generated one tissue or cell type at a time it is not always feasible to extend the results from one setting to a new cell type or point in development. It would be extremely useful to be able to scan for binding affinity across the complete transcriptome and to predict exon targets in tissues that have not yet been subjected to experimental analysis.

Splicing regulatory proteins commonly contain multiple RRM or other RNA binding domains, with each domain recognizing a short element of a few nucleotides [2], [16]. Subtle variation in the optimal binding element of each domain and flexible peptide linkers between them allow for significant degeneracy within high affinity binding sites. Although the short sequence motifs that are common to a set of binding sites are readily identified, these likely constitute only a portion of a full high affinity site. To rank binding sites and assess their finer structures, we need an approach to search for clusters of these short motifs and to score for binding affinity.

The Polypyrimidine tract binding protein 1 (PTBP1) is a widely studied splicing regulatory protein [17], [18]. PTBP1 is known to repress the splicing of a large number of exons by binding in their adjacent introns or within the exons themselves. PTBP1 is down regulated in differentiating neurons and muscle cells to allow inclusion of PTBP1 repressed exons during development of these tissues [19], [20], [21]. In neurons the loss of PTBP1 is accompanied by the up-regulation of the homologous protein PTBP2 [17], [20], [22]. PTBP2 has similar binding properties to PTBP1 and represses some of the same exons [23]. Other exons are more sensitive to PTBP1 than PTBP2 and are induced to splice when PTBP2 replaces PTBP1 in early neurons [24].

PTBP1 contains four RRM domains that recognize short pyrimidine elements [25]. Flexible linkers separate RRM domains one and two, and domains two and three. RRM domains three and four interact through a hydrophobic interface that position their RNA binding surfaces on opposite faces of the two-domain structure. This orientation requires that the RNA elements interacting with the structure be separated by an RNA loop [26]. The structure of each of the PTBP1 RRM domains has been solved in complex with the hexanucleotide, CUCUCU [25]. These structures show each domain binding a nucleotide triplet with some additional contacts, and making similar base specific interactions with CU or UC dinucleotides. Other sequences can likely make different base specific contacts, and the optimal elements for each domain are not known. Moreover, the flexible linkers separating some of the RRM domains and the requirement for a gap between elements simultaneously bound to domains three and four allow for substantial degeneracy in PTBP1 binding sites. This degeneracy and the lack of understanding of the sequence features that contribute to binding affinity have made it difficult to identify PTBP1 binding sites based on sequence alone, and to assess which sequences surrounding an exon might contribute to PTBP1 regulation.

Experiments with model substrates indicate that a single high affinity PTBP1 binding site placed upstream of an exon, or within it, can repress splicing [27]. However, strong repression of an efficiently spliced exon requires an additional binding site either within the exon or downstream from an exon with an upstream high affinity site [17], [27], [28]. PTBP1 is also known to enhance the splicing of certain exons [13], [19], [20]. The properties of these exons and how they differ from those that are repressed by PTBP1 are unclear, with different studies coming to different conclusions [13], [19]. An analysis of CLIP data in HeLa cells found that PTBP1 sites near the adjacent constitutive exons could enhance the inclusion of an alternative exon between them [13]. In contrast, examination of exons whose splicing was reduced by double knockdown of *Ptbp1* and *Ptbp2* found that they frequently had binding sites immediately downstream [19], whereas splicing repression often involved upstream binding sites: a pattern observed for other splicing regulators. These results are not mutually exclusive. It is possible that the two groups examined different subsets of the many exons regulated by PTBP1, and that the protein may show additional patterns of protein binding adjacent to its target exons.

In this study we sought to understand the sequence features that determine RNA binding by PTBP1 and to examine how they are combined in exons that are targeted by the protein. We first developed a statistical model of PTBP1 binding sites that identifies new features of RNA recognition by the protein. This binding model was then applied to the assessment of exon regulation by PTBP1 across the transcriptome.

## Results

### G containing triplets contribute to PTBP1 binding

**Figure 1:**
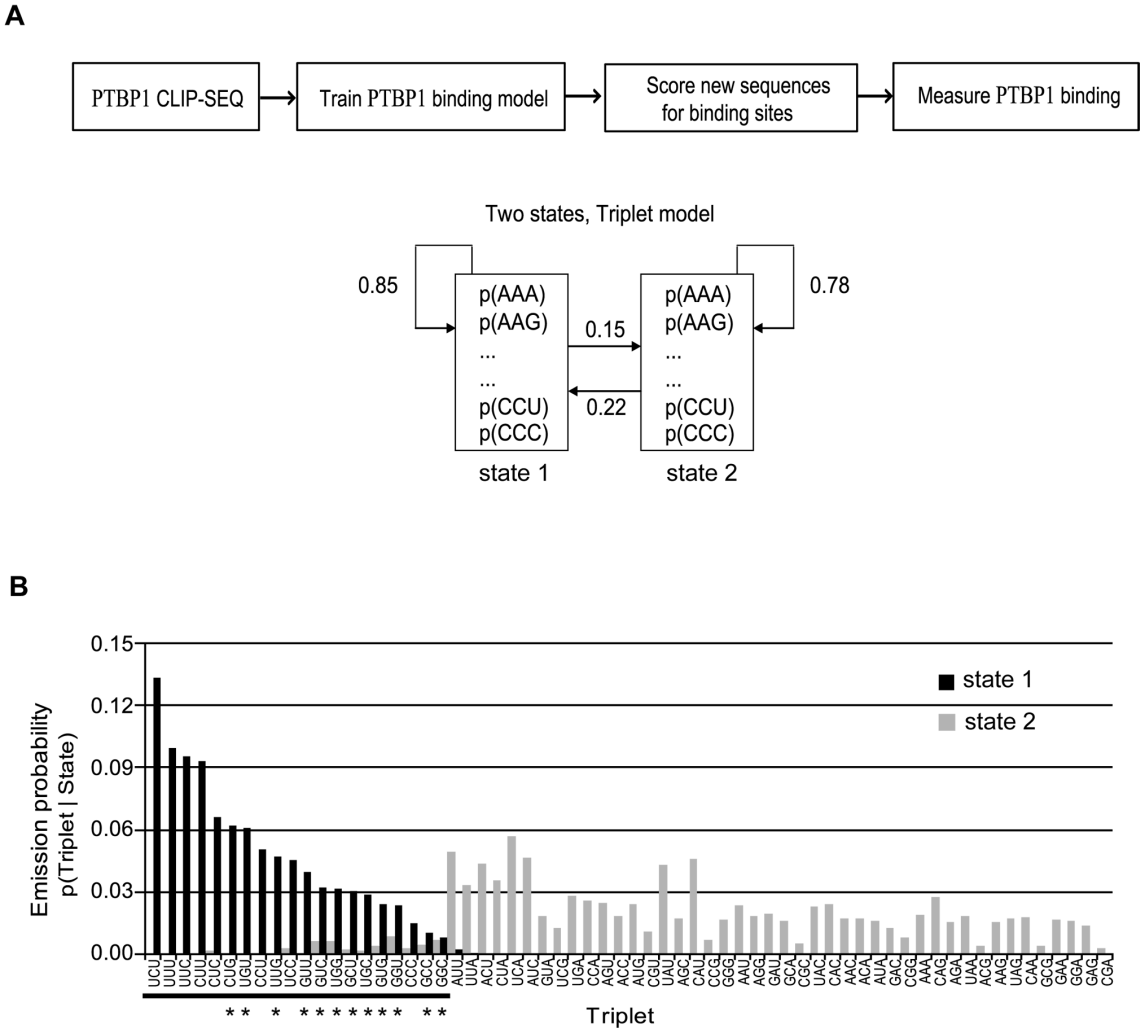
PTBP1 binding model. **A**. Scheme of the PTBP1 binding model. The two-state HMM model was trained on PTBP1 bound RNA sequences (48,604 clusters) from published PTBP1-CLIP experiments. Triplets from these CLIP clusters were predictive of two states, with all of the pyrimidine triplets preferred by State 1. The diagram presents the structure of the PTBP1 HMM (Hidden Markov Model) and its trained transition probabilities. **B**. The probabilities that triplets are seen states 1 or 2 (emission probabilities) are plotted in black and gray bars, respectively. Asterisks indicate G containing pyrimidine triplets.

To examine the interactions of PTBP1 across many binding sites, we used a set of PTBP1-bound sequences identified by crosslinking immunoprecipitation (CLIP) [13]. PTBP1 has four RRMs separated by linker peptides, with each RRM recognizing a pyrimidine triplet. In previous studies we found that a minimal high affinity binding site for the protein extended across 25 to 30 nucleotides, about the average size of the CLIP clusters (29 nt) [27]. Given the triplet recognition and the need for spacers between the direct RRM contacts, it is unlikely that every nucleotide within a CLIP cluster makes a direct base-specific contact with the protein or otherwise contributes to binding affinity. This information about direct binding is hidden in the examination of a CLIP tag, but should affect the triplet frequencies within the entire set of tags. We designed a two-state Hidden Markov Model (HMM) based on triplets to assess whether triplets would segregate into two states and whether these two states differed in their PTBP1 binding or non-binding potential. The 48,604 CLIP clusters from the human transcriptome were extracted and used to train the HMM (Figure 1A) [29], [30]. This training defined two states showing distinctly different triplet distributions (Figure 1B). Pleasingly, all of the pyrimidine triplets segregated into State 1. We called this state the PTBP1 binding state, as we confirm below. We found that 20 triplets have higher probabilities to be seen in the PTBP1 binding state. All triplets containing only pyrimidines were included in this 20-triplet set (Figure 1B), with the top-scoring triplet UCU showing the alternating C and U nucleotides seen in many characterized PTBP1 binding sites.

Interestingly, multiple triplets containing G residues are also preferred in State1 (Figure 1B). These triplets often contain U residues as the other nucleotides. Some of these triplets, such as UGU, have output (emission) probabilities in State 1 that are similar to pyrimidine triplets, presumably also making them predictive of PTBP1 binding. In contrast, triplets containing A residues, even if the other two nucleotides are pyrimidines, were all preferred by the non-PTBP1 binding State 2. These results indicate that PTBP1 is not strictly pyrimidine specific. At least one of its RRM domains can presumably make specific contacts with G residues. On the other hand, all A containing triplets have modest positive emission probabilities for state 2 and are likely to be either neutral or to inhibit PTBP1 binding.

We next tested the HMM scoring, which strongly weights the triplets from state 1 over state 2, for prediction of PTBP1 binding. We performed cross validation experiments on the Hela CLIP dataset. A background dataset was generated using ten randomly picked sequences from each gene identified as containing a CLIP cluster. Applying the model to this data set gave us a distribution of scores that was compared to scores generated by subsets of the CLIP clusters removed from the training set prior to training. As shown in Figure S1, sequences from subsets of the CLIP clusters scored significantly higher than background.

We also tested our model on an independent iCLIP dataset from human embryonic stem cells (ESC) (Figure S2). Unlike standard CLIP, iCLIP tags define the probable crosslink site as being the 5′ terminus of the tag. We used a Viterbi algorithm to predict the most probable state path predicted by the PTBP1 HMM model for each iCLIP tag. Defining triplets from the State1 (PTBP1 binding) and triplets from State 2 (nonbinding), we found that the frequency of predicted binding triplets is highly enriched in the iCLIP cluster regions and peaks precisely at the crosslink site. This indicates that State 1 probability is highly associated with PTBP1 crosslinking *in vivo*.

**Figure 2:**
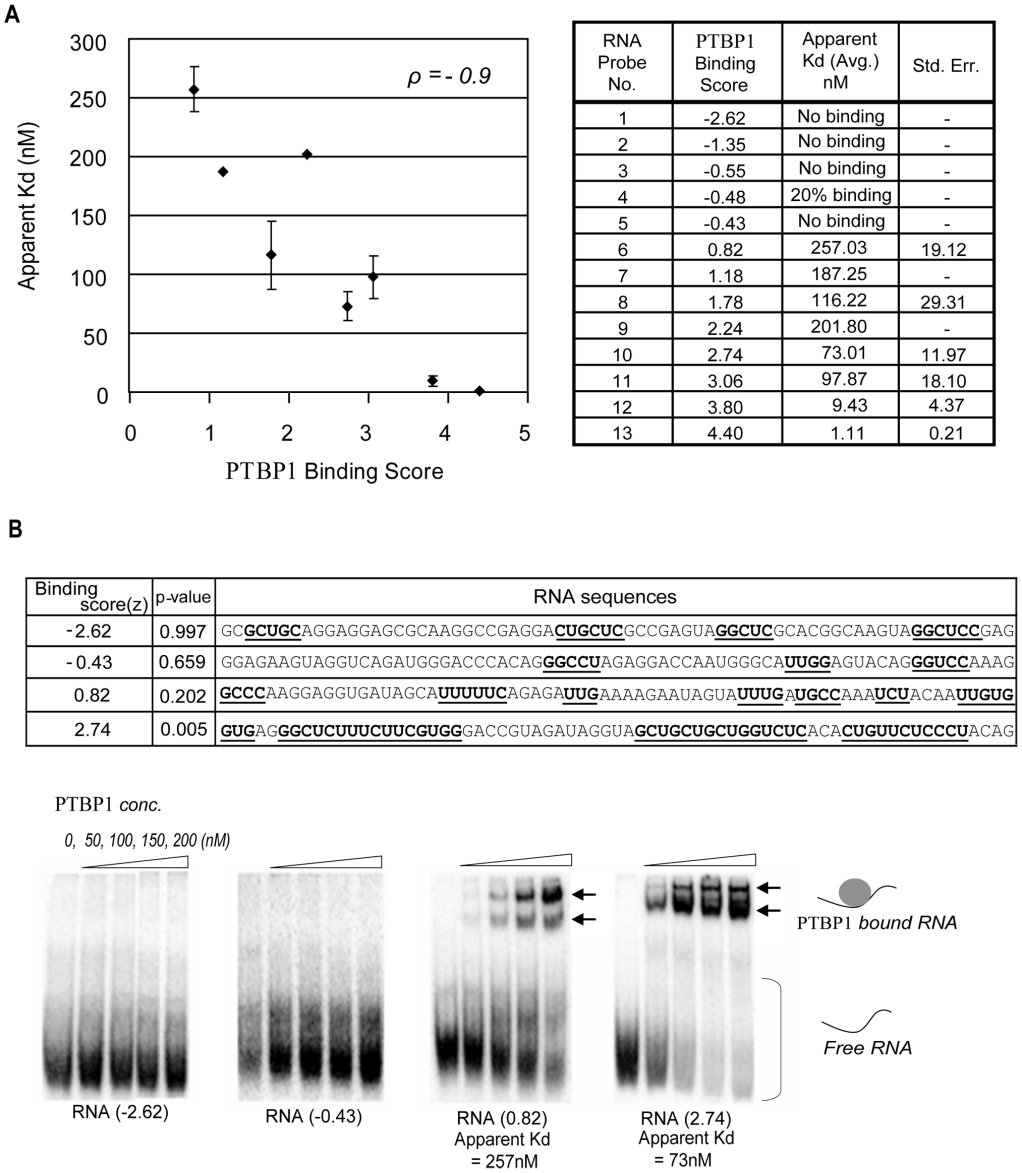
Validation of the PTBP1 binding model. **A**. To validate binding scores, thirteen RNAs with various PTBP1 binding scores were transcribed *in vitro* and subjected to binding assay. Apparent *Kd*'s (dissociation constant) were highly negatively correlated with PTBP1 binding scores (Pearson correlation = −0.9). **B**. Four RNA sequences with predicted PTBP1 binding scores (Full data binding data in Figure S3). Potential PTBP1 binding sites are underlined and in bold. Experimental binding affinities were assessed by electrophoretic mobility shift of RNA by PTBP1 and compared with prediction scores. Apparent dissociation constants (*Kd*) were defined as the concentration at which half the protein was bound to RNA.

To more quantitatively assess the relationship between the HMM score and RNA binding, we applied the trained model to a set of 100,000 random 69 nucleotide sequences. This length allows for one hexanucleotide binding site for each of the four RRMs with 15 nucleotide gaps, the minimum gap required for simultaneous binding by RRMs 3 and 4 [25], [26]. The scores are calculated as a log-odds ratio of the probabilities of the sequence having been generated by the HMM over a background model that assigns equal probability to all triplets. The random sequences generated a distribution of scores that was used to normalize the binding scores, with the average score for random sequence set to zero, and the z-score defined as the deviation from the average as shown in Figure S3A [29]. Thus a sequence with a z-score of 2.74 is 2.74 standard deviations from the average (empirical p-value = 0.005), and is predicted to be a significantly stronger binder than the average sequence (500 of the 100,000 random sequences have scores equal or greater than this sequence). A negative z-score is predicted to bind less well than the average sequence. We isolated thirteen sequences from the mouse transcriptome that exhibited a range of scores from −2.62 to +4.40 (Figure 2A). These were transcribed *in vitro* and subjected to electrophoretic mobility shift assay to measure binding to recombinant PTBP1 (Figure 2B; Figure S3B). Sequences yielding negative scores all failed to bind PTBP1 within the protein concentration range tested, with the exception of probe 4, which bound weakly, below the level that would allow measurement of an affinity constant. Positive scoring sequences all yielded PTBP1 bound complexes that were assayable by gel shift to derive apparent binding affinities. The apparent *Kd*s of these RNAs showed a very strong negative correlation with their binding score from the model (Pearson correlation coefficient = − 0.9), where a higher score predicts a lower *Kd* and hence a higher affinity (Figure 2A). Thus, the scoring system performed very well in predicting PTBP1 binding affinity.

Two sequences (probes 9 and 11) showed variable binding that shifted their *Kd*'s slightly off the fitted curve relating z-score to *Kd*. These may have secondary structures that reduce binding affinity thus increase their apparent *Kd*. To look at this, we examined the predicted structure of each probe using the RNA fold program [31]. Probes 9 and 11 did not show an overall free energy of folding substantially lower than other RNAs. However, it is difficult to rule out that they contain a local structure that sequesters some key feature for PTBP1 recognition.

In addition to the background model using uniform triplet frequencies, we also tested control sequence sets using different nucleotide frequencies (Figure S4). Control sets that maintain the mono or dinucleotide frequencies of the PTBP1 CLIP tags while shuffling the triplet frequencies did not perform well. This is not surprising because these sequences are highly skewed in nucleotide content and the shuffling does not change the triplet frequencies dramatically. We also tested a background model based on random sequences selected from genes containing PTBP1 CLIP clusters (ten sequences from each gene). Like the random dataset, this background model generated scores that predicted affinity reasonably well. However, it did generate negative scores for a couple of probes that are shown to bind (data not shown). Thus, the uniform model gave the most accurate scoring of the background models we tested.

The data demonstrate that HMM scoring based on triplet frequencies can accurately predict the observed binding affinities across a wide range of *Kd* values (from ∼250 nM to 1 nM). Probe 6 yields a z-score of 0.82 and binds with a *Kd* of 257 nM, whereas probe 10 scores 2.74 in the model and binds with a *Kd* of 73 nM (Figure 2B). These sequences include G containing triplets that contribute to the binding scores. This method allows any sequence to now be quantitatively assessed for possible PTBP1 binding, which was not previously possible by simply looking for clusters of a limited number of motifs. This HMM based approach should be applicable to the prediction of binding sites and affinity for other multi-domain RNA binding proteins.

### Placement of PTBP1 binding sites adjacent to target exons

With our new method of defining PTBP1 binding sites, we next examined PTBP1 target exons for the location of predicted PTBP1 binding. In part, we wanted to reassess two previous studies that came to differing conclusions regarding the placement of PTBP1 sites adjacent to its target exons. One group mapped PTBP1 CLIP clusters adjacent to a limited number of PTBP1 repressed and enhanced exons [13]. This study described PTBP1 repressed exons as enriched for binding sites both upstream and downstream, as has been seen in studies of individual exons. They did not observe PTBP1 CLIP clusters within repressed exons, even though such exons have been described [17], [32], [33]. The PTBP1 enhanced exons they examined showed a trend in PTBP1 binding near the flanking constitutive exons. A second study examined exons showing altered splicing on splicing-sensitive microarrays after *Ptbp1*/*Ptbp2* double knockdown [19]. CLIP clusters derived from the first study were mapped to these exons. The authors found CLIP cluster enrichment upstream and within PTBP1/PTBP2 repressed exons. In contrast to the previous study, they found that PTBP1/PTBP2 enhanced exons showed enrichment for CLIP tags in the downstream region. This pattern of binding site placement relative to repressed and enhanced exons has been observed for several other splicing regulatory proteins [14], [34].

**Figure 3:**
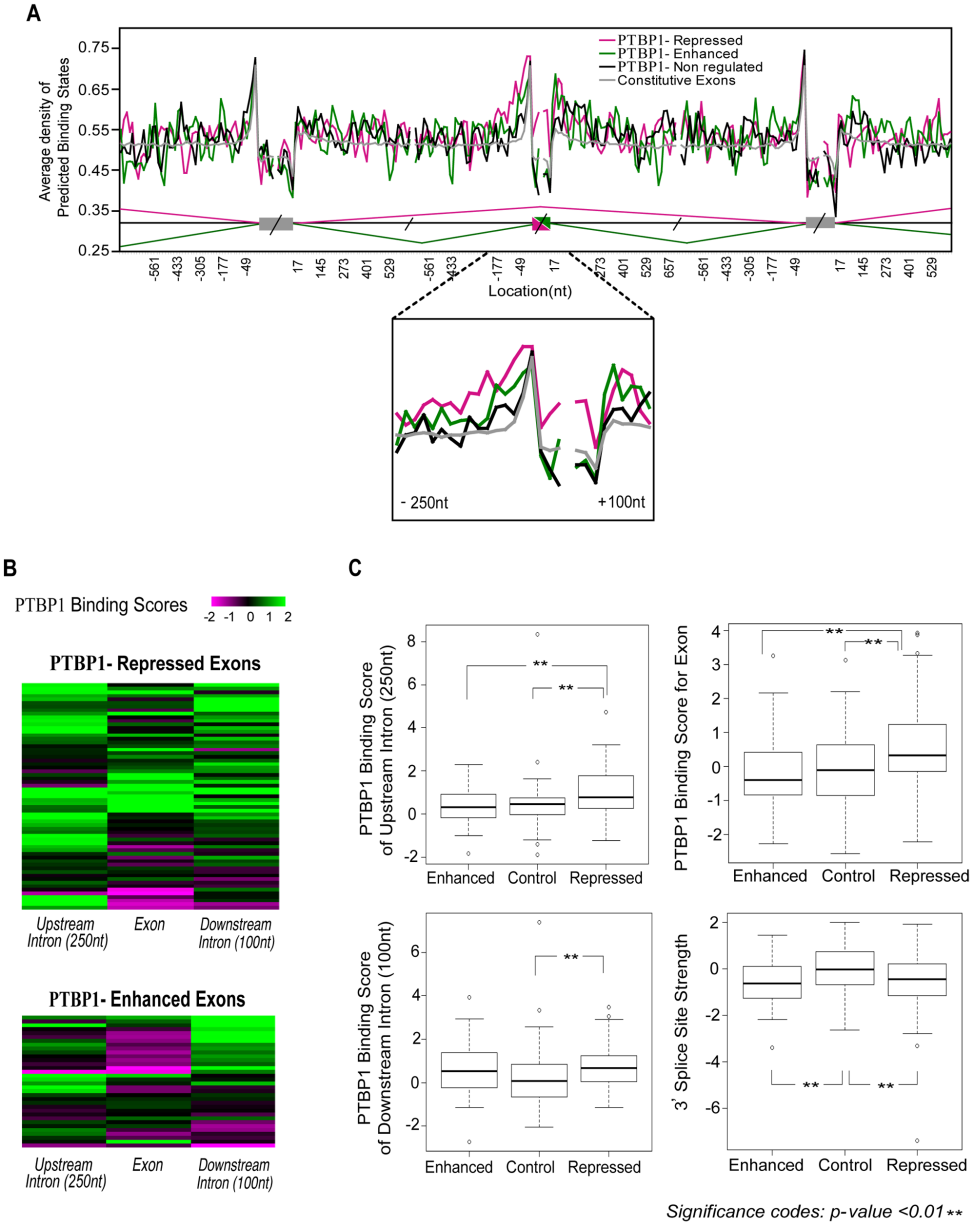
Sequence characteristics of PTBP1-dependent alternatively spliced exons. **A**. An RNA map shows enrichment of predicted PTBP1 binding sites near PTBP1-dependent exons. The Y-axis plots average density of predicted PTBP1 binding states within a 24 nt window; the length of overlap between two adjacent windows was 8 nt. **B**. To assess PTBP1 binding signatures of individual exons, known PTBP1 regulated exons were clustered by their PTBP1 binding score profiles and visualized as heat maps. These heat maps indicate wide variation in the positions of PTBP1 binding sites between individual exons. **C**. Four sequence features including the PTBP1 binding scores and 3′ splice site strength show statistically significant differences between regulated and control exon groups (one-tailed Student's t-tests).

In our study, we defined four groups of exons from a set of exons previously assessed for splicing after *Ptbp1* knockdown [20], [35]. These included 68 PTBP1-repressed exons whose splicing increases after *Ptbp1* knockdown, 37 PTBP1-enhanced exons whose splicing decreases after knockdown, 69 control exons that are not affected by *Ptbp1* depletion but are known to be alternatively spliced (PTBP1-non regulated), and 1,000 constitutive exons. We determined the density of predicted PTBP1 binding states within a 24-nucleotide window sliding along the exon region. We also examined the sequence encompassing the adjacent constitutive exons (Figure 3A). As expected, the non-regulated control and constitutive exon sets did not exhibit high probabilities of PTBP1 binding except in the polypyrimidine tract of the 3′ splice site. On the other hand, the introns upstream of PTBP1 repressed exons show enrichment of potential PTBP1 binding sites starting from 250 nucleotides upstream of the exon. Relative to the control exons, exons repressed by PTBP1 also exhibited substantial enrichment of PTBP1 binding sites within the exon itself and within the first 100 nucleotides of the downstream intron. The repressed exons thus exhibit binding site placement that combines the findings of the two previous studies [13], [19]. The PTBP1-enhanced exon set also shows enrichment of PTBP1 binding sites within the downstream intron relative to control exons, although the distribution of binding sites across this region was different between the repressed and enhanced exon sets (Figure 3A). Similar to what was seen in the previous study by Llorian, we found little enrichment of PTBP1 sites within enhanced exons [19]. There is a limited enrichment adjacent to the exons flanking enhanced exons. Interestingly however, we find some PTBP1 enhanced exons that have PTBP1 binding sites upstream of the exon. These were not seen in either previous study. Our results are generally consistent with the known placement of PTBP1 binding sites in PTBP1 target exons and imply that rules correlating the position of PTBP1 binding to its effect on a target exon are not as strict as seen for some other splicing regulators. The mechanisms proposed from previous maps of PTBP1 binding do not appear to be generalizable to all PTBP1 targets [13], [19], [27].

Binding maps for PTBP1 and other splicing regulators show the averages of multiple exons. Since the data indicated a high level of variability in binding site placement between individual exons, we wanted to visualize target exons relative to each other. To display binding signals for individual exons we created heat maps of the binding scores upstream, within, and downstream of each exon in the PTBP1 target set (Figure 3B). This display makes clear that the location of PTBP1 binding sites within its known target exons is variable. We found that 60% of PTBP1 repressed exons are predicted to have strong binding sites within the upstream intron. Most of these exons also have strong binding sites within either the exon or the downstream intron, patterns that were observed previously [13], [19], [27]. However, other patterns of binding site placement are also seen, suggesting PTBP1 dependent exons are following multiple rules. Some repressed exons score highly for PTBP1 binding only within the exon or in both the exon and the downstream intron. About half of PTBP1 enhanced exons have strong PTBP1 binding sites downstream (Figure 3B). These can co-occur with upstream intron-binding sites, but rarely with exon binding sites. Interestingly, there are exons enhanced by PTBP1 with strong upstream binding in the absence of other sites. These data demonstrate the heterogeneity in the position of PTBP1 binding sites for its target exons. This heterogeneity needs to be considered for predicting PTBP1 dependent regulation.

PTBP1 repressed exons exhibited significantly higher average binding scores in both the upstream intron and in the exon itself, than either the control group of alternative exons or the PTBP1 enhanced exons (Figure 3C). The average binding scores in the downstream introns were higher for both the PTBP1-repressed and PTBP1-enhanced exons than the control group (Figure 3C), although not at the same statistical significance. The variability of binding site placement within the smaller group of PTBP1-enhanced exons presumably contributes to the weaker statistical correlation of binding scores with positive regulation.

We also compared the three exon sets for other features that might contribute to their ability to be regulated by PTBP1, including exon length, flanking intron length, and 5′ and 3′ splice site strength. Most of these features were not statistically different among the three-exon groups. However, both PTBP1 enhanced and PTBP1 repressed exons were found to carry significantly weaker 3′ splice sites than the control exon set, as measured by the Analyzer Splice Tool (Figure 3C) [36], [37].

These results indicate that PTBP1-repressed exons, and perhaps PTBP1-enhanced exons, exhibit an ensemble of sequence features that define them as PTBP1 regulated and that should allow their identification by sequence alone.

### Prediction of PTBP1 repressed exons

Alternative exons are generally regulated by multiple factors that act both positively and negatively on their ability to be spliced. Thus, an exon controlled by a regulator in one context might not be affected by it under other conditions where counteracting factors are present, or required cofactors are absent. This means that the most accurate predictions of splicing regulation will need to consider many different factors. Nevertheless, models based on single factors will be useful for understanding the relative contributions of individual proteins to patterns of splicing regulation. Such models will be easier to interpret regarding the contributions of individual factors to individual exons than more complex models. Moreover in the longer term, models developed for different individual factors can be combined to make more accurate predictions. To assess how well one might model splicing regulation by a single factor, we examined whether the strength and placement of predicted PTBP1 binding sites could be used to predict new PTBP1 dependent exons. We plotted the scores for a variety of sequence features against the percent of exons exhibiting that score that also exhibit PTBP1 dependent exon repression (Figure S5). These plots produced distinct sigmoidal curves where most exons regulated by PTBP1 were found above or below a particular score. This strongly suggests that a logistic regression model incorporating each of these scores will be predictive of PTBP1 repression.

**Figure 4:**
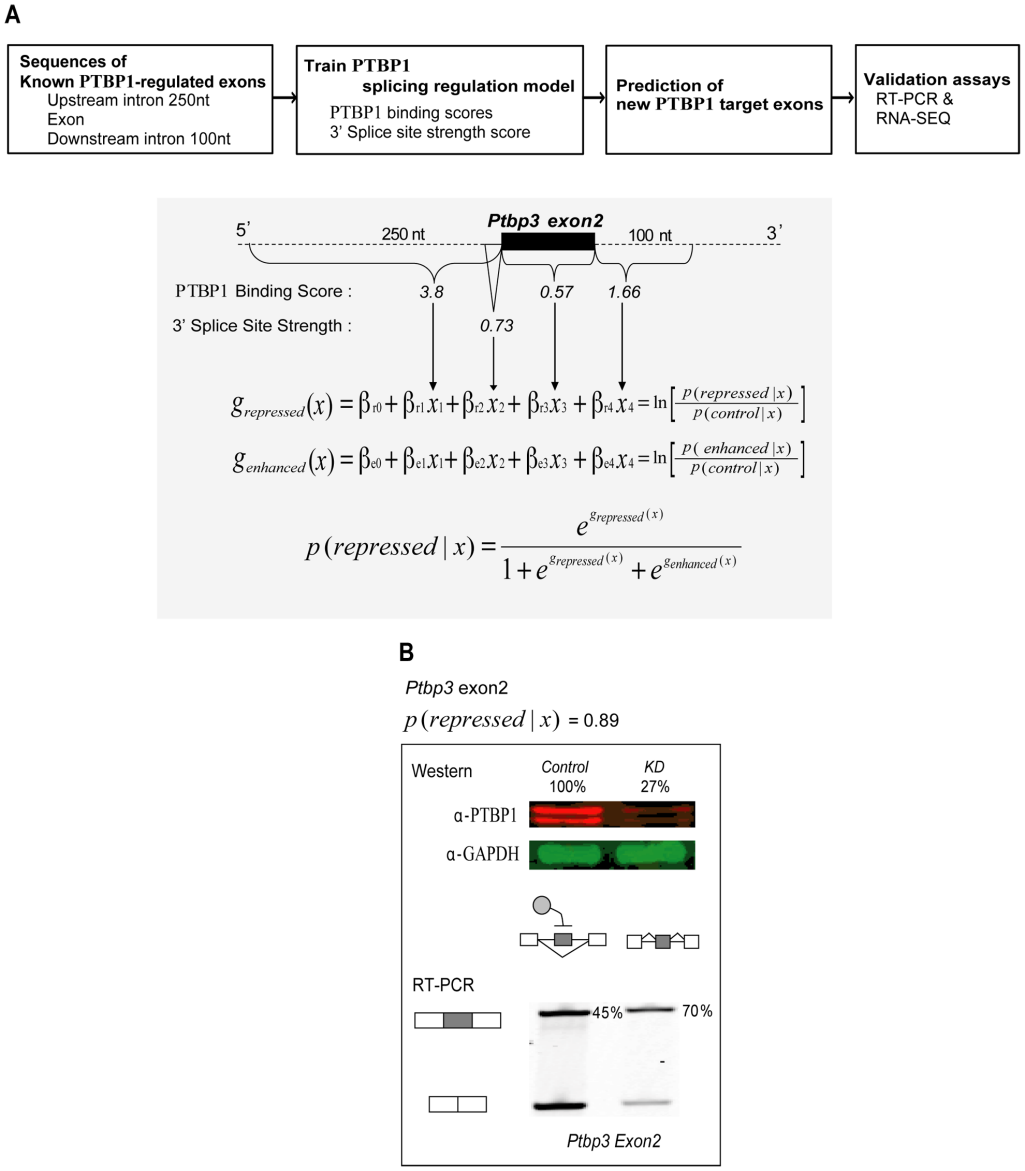
Scheme of the PTBP1 splicing regulation model and its application to an exon in *Ptbp3*. **A**. The PTBP1 splicing regulation model was trained on known PTBP1-regulated and non-regulated exons and used to predict new PTBP1-dependent exons. Prediction results were compared to changes in exon inclusion (PSI) measured by RT-PCR and RNA-seq. An exon from *Ptbp3* is presented as a prediction example. From intron and exon sequences, PTBP1 binding scores and 3′ splice site strength were calculated and fed into the regulation model. **B**. The model predicts exon 2 of *Ptbp3* as repressed by PTBP1 with high probability (0.89). *Ptbp1* knockdown in mouse neuroblastoma cells (*N2A*) confirmed de-repression of the exon (from PSI = 45 to PSI = 70).

We developed a multinomial logistic regression model and trained it on three classes of regulated exons (Figure 4A) [38]. The training set included PTBP1 repressed exons, PTBP1 enhanced exons, and non-regulated exons. Each exon in each class was scored for the four features found to correlate with PTBP1 regulation (*x*_1_ through *x*_4_), including the 3′ splice site strength, and the PTBP1 binding scores for each of three regions: the 250 nucleotides upstream of the exon, the exon itself, and the 100 nucleotides downstream of the exon. These intron lengths encompass the regions of binding site enrichment for PTBP1 dependent exons (Figure 3).

The PTBP1-enhanced exons are fewer in number and show more limited enrichment of PTBP1 binding sites than PTBP1-repressed exons making the prediction for these exons less accurate. We first tested models that considered just PTBP1-repressed exons relative to control groups. However, we found that including the enhanced exons as a separate training group improved the prediction of repressed exons, even though enhanced exons themselves are not as easily identified (data not shown).

The trained model yielded values for the β coefficients that weight the different features contributing to the regulation. As expected the upstream binding score was weighted most heavily in predicting PTBP1 repression (Table S1), although binding scores in all three regions contributed to the score for PTBP1 repression. In contrast, we found that only the downstream binding score was significantly associated with PTBP1 enhancement. The upstream score generated a β coefficient close to zero making it essentially neutral in the prediction of enhanced exons. The exon binding score was subject to a negative β coefficient, indicating that exon binding reduces the probability of PTBP1 enhancement. Using these β coefficients, the trained models for repression or enhancement each yield a value of the g-function (logit) for an exon (*x*) given by the log of the ratio of the probability of repression or enhancement over the probability that the exon is not regulated. From this, the probability that an exon is repressed by PTBP1 can be determined from the two g-values as shown in Figure 4A.

We assessed the multinomial logistic regression model by recursively retraining on exon sets with one exon left out and then scoring the missing exon. This leave-one-out cross validation enabled assessment of the overall performance of the model [38] (Figure S6). The PTBP1 dependent exon repression logit showed good prediction, with an area under the curve (AUC) value of 0.72, substantially greater than random guessing (AUC = 0.5). As expected, the enhanced exon logit was not as accurate as the repression logit (AUC = 0.57), although it was better than random (Figure S6A). Using these data, we assessed the sensitivity and specificity across the range of scores to define a decision threshold for exon repression scores (Figure S6B). Increasing the threshold increases the specificity by eliminating many false positives, but decreases the sensitivity of the model in identifying maximum numbers of repressed exons. We sought to choose a threshold that gave a low false positive rate over one that yielded more regulated exons. We found that above a threshold score of 0.65 the false positive rate was 10% or lower (Figure S6B).

**Figure 5:**
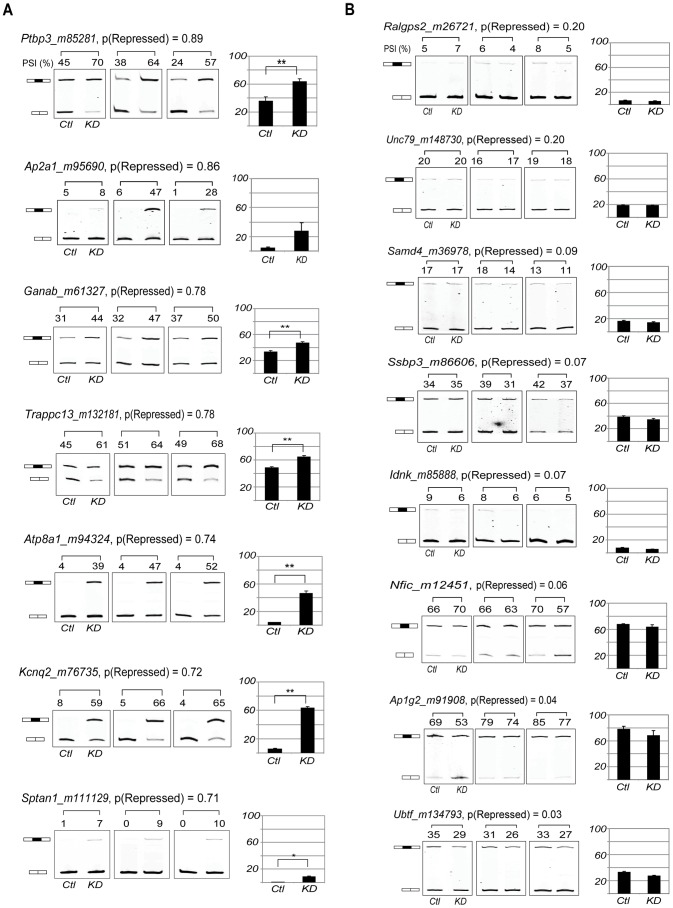
Validation of novel PTBP1-repressed exons by RT-PCR. **A**. Candidate PTBP1-repressed exons with probability greater than 0.65 were validated by RT-PCR following *Ptbp1* knockdown. Data shown are averages ± standard error of PSI (Percent of Spliced In) from biological triplicates. Statistical analysis was performed using paired one-tailed Student's t-test (p-values<0.01**, <0.05*). **B**. Exons with low PTBP1 repression probabilities (≤0.2) were also validated by RT-PCR following *Ptbp1* knockdown in biological triplicates.

Applying the model to 4494 alternative cassette exons from UCSC genome browser database, we found 243 exons (5.4%) that yielded a PTBP1 repression probability score greater than 0.65 and which were not in the training set. The 50 top-scoring cassette exons are listed in Table 1. These included two exons that were reported previously to be PTBP1 targets. An exon of *Gabrg2* yields a probability score of 0.92. Although we could not confirm its repression in N2A cells because of low expression of the transcript, the orthologous exon in rat is a well-characterized PTBP1 repression target [39]. Exon 2 of *Ptbp3 (Rod1)*, another known PTBP1 target [40], yielded a repression probability score of 0.89 and was confirmed by RT/PCR to show increased inclusion after *Ptbp1* knockdown (Figure 4B). We performed additional RT-PCR validation in triplicate on a series of high and low scoring exons from transcripts expressed in N2A cells (Figure 5 & Figures S7, S8 and S9). Seven of ten exons scoring above 0.65 were de-repressed after *Ptbp1* knockdown in N2A cells, yielding a validation rate of 70%. The actual false positive rate is difficult to estimate because exons with high repression scores that are not affected by *Ptbp1* depletion in N2A cells might be regulated by PTBP1 in other cells. An indication that this might be occurring is that the average inclusion level (or percent spliced in value, PSI) of the putative false positives is significantly higher than the confirmed true positives in N2A cells, indicating that they will be less prone to change upon *Ptbp1* depletion and be more difficult to validate (Figure S8B). Thus, the true positive rate may be greater than 70%. Importantly, the high validation rate for exons scoring above 0.65 indicates that the binding model and the regulation model based upon it can identify many new PTBP1 targets that were not previously known (Table1).

**Table 1:** PTBP1 repressed exons identified by the splicing model.

Gene Name	Gene Description	mm9 coordinates	PTB Binding Scores			3′	p(Repressed)
			Upstream		Downstream	Splice site	
			Intron (250 nt)	Exon	Intron (100 nt)	Strength	
*Pax6*	paired box gene 6	chr2:105523985–105524115(+)	8.35	−1.49	−0.80	0.27	0.99
*Mbd5*	methyl-CpG binding domain protein 5	chr2:49134101–49135303(+)	6.46	−0.93	2.27	−0.32	0.98
*Arhgap24*	Rho GTPase activating protein 24	chr5:102981145–102981338(+)	6.47	0.10	−0.05	0.46	0.97
*Tle1*	transducin-like enhancer of split 1	chr4:71819247–71819451(−)	4.71	0.05	−1.26	−2.56	0.94
*Acsl6*	acyl-CoA synthetase long-chain family	chr11:54150438–54150515(+)	4.16	1.40	−0.03	−0.82	0.94
*Ryr1*	ryanodine receptor 1, skeletal muscle	chr7:29829938–29829955(−)	4.78	−0.08	−0.88	−1.71	0.94
*Ankhd1*	ankyrin repeat and KH domain containing 1	chr18:36784163–36784921(+)	4.37	0.03	1.44	−0.64	0.93
*Slc39a14*	solute carrier family 39 (zinc transporter)	chr14:70713408–70713577(−)	3.51	1.16	−1.27	−3.20	0.92
*Gabrg2*	gamma-aminobutyric acid (GABA) A receptor	chr11:41727472–41727495(−)	1.95	2.77	0.56	−3.79	0.92
*Itga7*	integrin alpha 7	chr10:128378878–128378997(+)	4.14	0.29	1.13	−0.25	0.92
*Iqsec2*	IQ motif and Sec7 domain 2	chrX:148615540–148615635(+)	4.88	0.68	−0.13	0.71	0.91
*Smarca2*	SWI/SNF related, matrix associated, actin dependent regulator of chromatin	chr19:26825612–26825646(+)	3.94	−0.09	1.23	−0.86	0.91
*Zfand3*	zinc finger, AN1-type domain 3	chr17:30197755–30197795(+)	4.17	2.34	0.90	1.80	0.91
*Agap2*	ArfGAP with GTPase domain, ankyrin repeat and PH domain 2	chr10:126527198–126527257(+)	3.57	0.06	−0.53	−3.08	0.90
*Ttn*	Titin	chr2:76723554–76723832(−)	2.93	1.06	1.19	−1.83	0.90
*Ptbp3*	ROD1 regulator of differentiation 1 (S. pombe)	chr4:59559021–59559054(−)	3.80	0.57	1.66	0.73	0.89
*Mapk8*	mitogen-activated protein kinase 8	chr14:34203859–34203930(−)	2.35	1.17	1.01	−3.48	0.89
*Snap91*	synaptosomal-associated protein 91	chr9:86693373–86693534(−)	2.60	1.89	−0.35	−2.17	0.88
*Fmnl1*	formin-like 1	chr11:103059449–103059547(+)	3.93	−0.60	−1.36	−2.70	0.88
*Phldb1*	pleckstrin homology-like domain, family B	chr9:44509029–44509169(−)	3.20	0.57	1.05	−0.66	0.87
*2310035C23Rik*	RIKEN cDNA 2310035C23 gene	chr1:107637012–107637094(+)	2.03	1.76	0.85	−2.46	0.87
*Arnt*	aryl hydrocarbon receptor nuclear translocator	chr3:95270715–95270759(+)	3.48	−0.36	2.53	0.09	0.87
*Smyd2*	SET and MYND domain containing 2	chr1:191723697–191723807(−)	3.33	0.33	−0.28	−1.64	0.86
*Ap2a1*	adaptor protein complex AP-2, alpha 1 subunit	chr7:52158832–52158897(−)	3.35	−0.12	−0.89	−2.69	0.86
*Klra*	killer cell lectin-like receptor, subfamily A	chr6:130329011–130329100(−)	2.82	3.18	0.62	0.77	0.86
*Spag9*	sperm associated antigen 9	chr11: 93942054–93942068(+)	0.99	3.01	1.62	−3.03	0.86
*Col4a3bp*	collagen, type IV, alpha 3 binding protein	chr13:97386949–97387026(+)	2.81	1.23	0.74	−0.93	0.86
*Garnl3*	GTPase activating RANGAP domain-like 3	chr2:32941395–32941464(−)	4.15	0.38	0.36	0.94	0.86
*Dennd1a*	DENN/MADD domain containing 1A	chr2:37982049–37982168(−)	3.37	0.80	1.35	0.59	0.86
*Ms4a7*	membrane-spanning 4-domains, subfamily A	chr19:11400297–11400353(−)	2.79	2.35	0.37	−0.05	0.86
*BC030307*	cDNA sequence BC030307	chr10:86169981–86170089(+)	2.75	−0.16	1.95	−2.40	0.85
*Phactr1*	phosphatase and actin regulator 1	chr13:43154940–43155146(+)	2.73	1.25	0.50	−0.97	0.85
*R3hdm2*	R3H domain containing 2	chr10:126902187–126902240(+)	1.66	1.98	1.96	−1.57	0.84
*Cdc14b*	CDC14 cell division cycle 14B	chr13:64306579–64306725(−)	1.42	2.75	2.44	−0.58	0.84
*Ubqln1*	ubiquilin 1	chr13:58282183–58282266(−)	2.88	0.98	−0.06	−1.17	0.84
*Ttn*	Titin	chr2:76739898–76740179(−)	2.63	−0.07	1.49	−2.38	0.84
*Stx3*	syntaxin 3	chr19:11857290–11857400(−)	3.00	−1.12	2.26	−3.62	0.84
*Slc8a3*	solute (sodium/calcium) carrier family 8	chr12: 82310340–82310458(−)	1.84	1.25	1.76	−2.25	0.84
*Zfp62*	zinc finger protein 62	chr11:49028057–49028156(+)	3.27	1.98	−0.51	0.19	0.83
*Dlg1*	discs, large homolog 1 (Drosophila)	chr16:31771843–31771941(+)	1.53	1.98	1.87	−1.65	0.83
*Nrxn2*	neurexin II	chr19:6463824–6463847(+)	3.35	−1.37	1.33	−2.26	0.83
*Klra7*	killer cell lectin-like receptor, subfamily A	chr6:130179953–130180042(−)	2.68	2.11	−0.39	−0.63	0.83
*Picalm*	phosphatidylinositol binding clathrin assembly	chr7:97330729–97330878(+)	1.15	2.15	2.30	−2.37	0.83
*Acad8*	acyl-Coenzyme A dehydrogenase family	chr9:26798168–26798277(−)	2.61	0.88	−0.31	−1.86	0.83
*Epn1*	epsin 1	chr7:5033620–5033723(+)	3.92	0.65	0.07	1.06	0.82
*Grip1*	glutamate receptor interacting protein 1	chr10:119422530–119422685(+)	2.66	−0.74	2.61	−3.13	0.82
*Csmd3*	CUB and Sushi multiple domains 3	chr15:47587514–47587627(−)	2.42	2.16	0.20	−0.45	0.82
*Lrrfip1*	leucine rich repeat (in FLII) interacting protein 1	chr1:92990137–92990214(+)	2.02	0.40	3.44	−2.21	0.82
*Srsf11*	serine/arginine-rich splicing factor 11	chr3:157703405–157703586(+)	1.09	2.05	−0.56	−4.75	0.82
*Tmem209*	transmembrane protein 209	chr6:30441087–30441184(−)	3.82	0.16	0.10	0.64	0.82

The 50 highest scoring exons predicted to be repressed by PTBP1 based on sequence alone.

High scoring exons might also fail to be validated because of regulation by other proteins. Knockdown of *Ptbp1* induces expression of its close homolog *Ptbp2*, which targets some of the same exons [20] (Figure S7). To test whether PTBP2 was also targeting the predicted PTBP1 repressed exons, we knocked down *Ptbp2* or both *Ptbp1* and *Ptbp2* expression in N2A cells and re-assayed the exons in triplicate (Figures S10, S11 & S8A). Although some exons showed greater inclusion in the double knockdown compared to depletion of *Ptbp1* alone, this did not validate any additional predicted PTBP1 repressed exons. We did identify some high and low scoring exons showing more complex regulation by the two PTB proteins (Figure S10 & S11).

We also examined a set of low scoring exons (probability score≤0.2) by RT-PCR after *Ptbp1* and/or *Ptbp2* depletion (Figure 5B and Figure S11). All of these exons (8 of 8) failed to respond to the loss of PTBP1 and are likely true negatives. Thus, PTBP1 repression scores above 0.65 and below 0.2 were highly predictive for regulation and its absence, respectively. As expected, intermediate scores were less consistent in their predictive value (Figure S9). Some exons in the intermediate scoring group were affected by PTB proteins and will be interesting to assess further.

The prediction of PTBP1-repressed exons was improved by treating PTBP1-enhanced exons as a separate class, but the probability scores for PTBP1 enhancement did not consistently identify new PTBP1 target exons (data not shown). This is likely in part due to the smaller number of exons in the training set and their heterogeneity, with some possibly being indirect targets. These predictions will likely improve with training on larger numbers of PTBP1 enhanced exons as they are identified. However, it is possible that simply the presence of the PTBP1 binding site is not sufficient for predicting PTBP1 enhancement and that binding sites for other factors will need to be considered.

We next tested the model on a genomewide scale, by applying it to a set of 168,111 mouse internal exons and ranking them by their probability of PTBP1 repression. This analysis yielded 3824 exons (2.3%) with probability scores above 0.65 for being repressed by PTBP1. Among other activities, these exons were enriched in genes that function in calcium ion transport, cytoskeletal organization, intracellular transport, and synaptic transmission, all functions affected by previously known PTB targets (Table S2).

**Figure 6:**
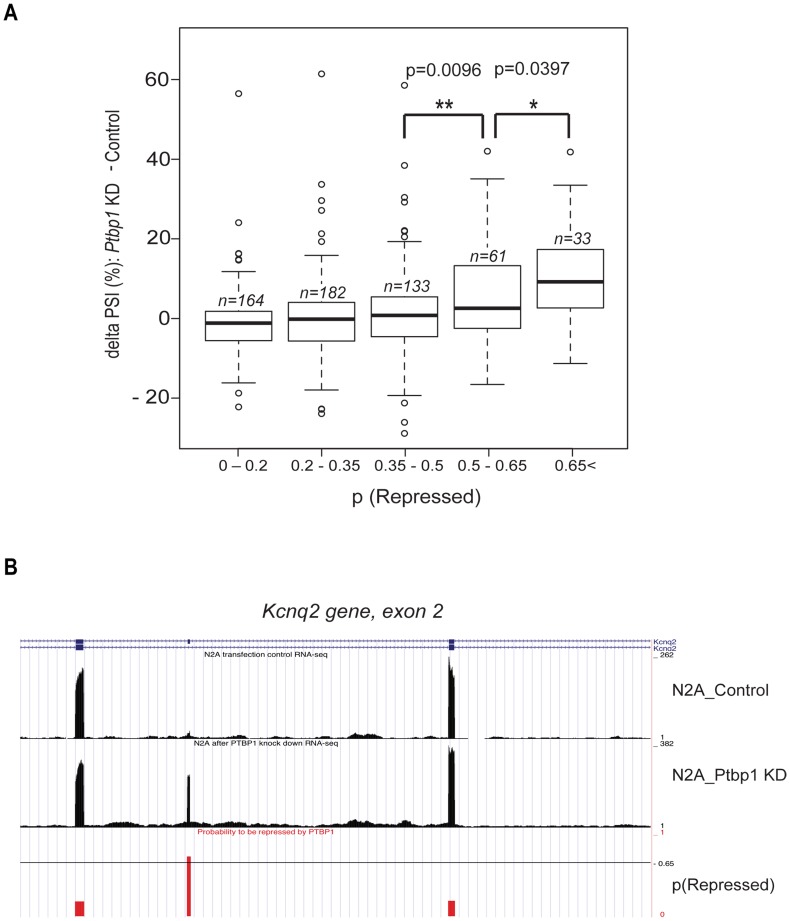
Large-scale validation of novel PTBP1-repressed exons by RNA-seq. **A**. Validation of the PTBP1 splicing model using RNA-seq. After *Ptbp1* knockdown, we performed RNA-seq experiments and estimated changes in PSI (Percent of Spliced In) for 573 cassette exons. The graph shows average delta PSI values for exons, grouped by their probabilities to be repressed by PTBP1. The number of exons in the corresponding probability bin is given by n. P-values were calculated from one-tailed Student's t-test. **B**. A genome browser screenshot of a novel PTBP1-regulated exon: exon 2 of the *Kcnq2* gene. For whole internal mouse exons, we created custom genome browser tracks to visualize the PTBP1 splicing model and mapped RNA seq reads.

To assess splicing of this large set of predicted PTB targets, we used RNA-seq to generate a large dataset of exons that change after *Ptbp1* knockdown. RNA from control and PTBP1-depleted N2A cells was subjected to high density short read sequencing on the Illumina HiSeq platform using a strand specific, paired end protocol [41]. Exons whose inclusion changed between the two samples were identified by alignment to an exon database and quantification of exon inclusion using the SpliceTrap program [42]. After filtering for read coverage and removing the training set, we identified 573 alternative exons whose splicing was assayable in N2A cells. These exons exhibit changes in percent exon inclusion (delta PSI) ranging from −29% to 62% upon PTBP1 depletion. The exons were binned by their PTBP1 repression probability scores and plotted for their change in PSI (Figure 6). The average changes in splicing were significantly correlated with the repression probability. Exons scoring below 0.5 distributed around zero change in PSI, but above this score the average exon inclusion is altered by PTBP1 depletion. Most notably, exons with a repression probability score above 0.65 exhibited significantly larger changes in splicing than exons with lower scores. Exons with intermediate scores and hence weaker binding sites show smaller changes in splicing than high scoring exons. Setting a threshold of a 5% change in PSI as validation, 22 of 33 exons (67%) that scored above 0.65 for PTBP1 regulation were confirmed as PTBP1 repression targets in N2A cells. At least some of the other 11 exons are presumably PTBP1 targets in other cells.

To test the model in another cell type, we examined exons reported to change after *Ptbp1* knockdown in mouse C2C12 myoblasts, as measured on splicing sensitive microarrays [43]. Very similar to what was observed in N2A cells, we found that exons with high repression probabilities showed significant de-repression upon the *Ptbp1* knockdown compared to exons with low repression probabilities (Figure S12). Of 29 exons assayed on the arrays with a repression probability above 0.65, 19 exons were confirmed as PTBP1 repressed on the array (q-value<0.05), yielding a validation rate of 66%. Thus the model performed very similarly in C2C12 and N2A cells. Among the 11 high scoring exons identified as unchanged after PTBP1 knockdown in N2A cells only 3 were assayed on the array and expressed in C2C12 cells. These again showed high inclusion in C2C12 prior to knockdown and so were difficult to assay for derepression. Thus, it is difficult to use the C2C12 data to draw conclusions about the false positive rate.

The logistical model gives us a new tool for studying the regulation of alternative splicing. Using it, we can now scan genomic sequence to score exons for PTBP1 regulation. Applying the model genomewide, the PTBP1 repression probability scores were integrated into the UCSC genome browser. These data, displayed with the RNAseq data from N2A cells are available at our website (http://www.mimg.ucla.edu/faculty/black/ptbatweb/). A novel PTBP1 repressed exon in the *Kcnq2* gene is shown in Figure 6B. The logistic model thus allows the assessment of any exon across the transcriptome for likely PTBP1 regulation.

## Discussion

### New features of PTBP1 binding sites

We have developed two computational models, one that allows accurate prediction of PTBP1 binding sites and another that predicts likelihood of PTBP1 repression of exons across the transcriptome. These models uncovered several new features of RNA recognition by PTBP1 and the properties of its target exons. The PTBP1 binding model was based on triplets following the structures of the PTBP1 RRM domains, whose sequence specific contacts are each primarily to three nucleotides. We find that the set of triplets that increase the probability of binding includes the expected pyrimidine motifs, particularly those with alternating cytosines and uridines. However, many triplets with guanosine residues also increase binding probability. In contrast, adenosine residues have a negative effect on binding. Thus, RNA recognition by PTBP1 is not solely dependent on pyrimidine nucleotides. The recognition of G residues by PTB was unexpected, although some previously characterized PTB binding sites did contain G residues [13], [44]. With this model, we can now predict PTBP1 binding affinity to any site in the transcriptome.

The base-specific contacts that PTBP1 makes with Guanosine are not yet clear. Recent studies of RNA recognition by SRSF2 (SC35) protein have shown that the element GGAG can be recognized by the same RRM as CCAG by flipping the initial two G nucleotides to the syn conformation [45]. It will be very interesting to investigate whether a similar anti to syn switch occurs in RNA bound by PTBP1, when C residues are replaced with G.

Previous characterizations of PTBP1 binding sites have focused on finding enriched short motifs within populations of bound RNAs or regulated exon sequences [13], [44], [46], [47], [48]. These methods generally identify elements whose short length will allow interaction with only one RRM domain. Searching for new binding sites comprised of clusters of these short elements can identify higher affinity sites but does not consider all elements or rank them. Crosslinking-immunoprecipitation experiments allow large numbers of binding regions to be identified. However, not all the sequence within a CLIP tag will be contacting the protein and it is difficult to relate CLIP signals to binding affinity. The HMM allowed the individual assessment of different short elements within the CLIP clusters, showing that they segregated into two states. The ranking of the triplets for their contributions to one of these states yielded a model where complex clusters of short elements could be assessed for binding and yielded accurate predictions of binding affinity. Many RNA binding proteins are similar to PTBP1 in having multiple domains that may each make different base specific contacts with RNA. The widespread generation of CLIP-seq datasets will allow the modeling of RNA recognition by almost any protein based on a large number of known binding sites.

Using the same modeling approach, we also developed a binding model for PTBP2 (neuronal PTB) using a published PTBP2 CLIP dataset [49]. PTBP2 is about 70% identical to PTBP1 in sequence, and has only two amino acid changes among the residues making direct contact with RNA [17]. We found that the binding models for two PTB proteins were also nearly identical indicating that the two proteins are likely to differ more in their protein/protein interactions than in their RNA binding sites (Data not shown).

### Defining PTBP1 target exons

Several PTBP1 target exons have been analyzed in detail [17], [50]. These exons vary in the placement and action of their PTBP1 binding sites. It is common for PTBP1-repressed exons to have a binding site upstream, often encompassing the branch point of the 3′ splice site [39]. Exons can also be repressed by PTBP1 binding within the exon [19], [32], [33]. Other exons contain downstream binding sites that are needed in conjunction with an upstream site to achieve splicing repression [51], [52], [53]. Although acting as a repressor for most of its targets, PTBP1 also activates the splicing of a group of exons. There have been divergent reports about placement of PTBP1 binding sites needed to mediate PTBP1 enhancement of splicing. The PTBP1 binding model allowed us to examine PTBP1 binding site placement across a large set of known PTBP1 target exons. Nearly all exons had predicted high affinity PTBP1 binding sites nearby. We found that more than half of PTBP1 repressed exons have high affinity binding sites upstream, and a fraction of PTBP1 enhanced exons have high affinity sites downstream. These exons fit with recent results on several other splicing regulators where the placement of the binding site determines the direction of the regulatory effect [12], [14], [34]. However, for PTBP1 these rules are not so clear. Some PTBP1 repressed exons have their strongest predicted binding site downstream or within the exon. These results indicate that there are fundamental differences between the mechanisms of PTBP1 mediated splicing regulation, and those governing regulation by certain other splicing factors.

To quantify the predictive value of the PTBP1 binding scores for PTBP1 repression, we built a logistic model for PTBP1 regulation. For exons repressed by PTBP1, binding scores for the upstream, downstream and exon sequences all contribute to the probability of repression. Exons enhanced by PTBP1 were too few to achieve accurate predictions from the model. However, treating these as a separate exon class improves the prediction of PTBP1 repression. We find that for probability scores above 0.65 the model is strongly predictive of PTBP1 repression. Applying this criterion across the transcriptome, we identified hundreds of new PTBP1 target exons.

Alternative exons are generally regulated by multiple proteins acting in combination, and a particular exon will often be subject to both positive and negative regulation by antagonistic factors. For a model based on one factor, these other proteins will confound predictions. Exons with high PTBP1 binding scores may be counteracted by antagonistic factors in some cell types. Alternatively, synergistic factors may allow an exon with a relatively weak binding site to still recruit PTBP1. Thus, a model based on one factor will be limited in its predictive power. In this study, our intent was to measure the effect of PTBP1 binding alone before considering the contributions of other factors. The logistic modeling allowed the contributions of different binding site placements to PTBP1 regulation to be measured.

Several studies have used Bayesian models to dissect the regulatory properties of exons [7], [54]. These models can generate accurate predictions by incorporating a wide variety of sequence, expression and conservation data. However, because so many disparate variables are incorporated, it can be difficult to draw mechanistic conclusions from these models regarding any one protein. For example, the presence of high pyrimidine density upstream from the branch point can be predictive of exons showing neuronal specific inclusion [7], [55]. This is presumably in part due to many neuronal exons being regulated by PTBP1 and PTBP2. However, a subset of these exons may be regulated by other factors with pyrimidine rich binding sites. In the long term, it will be most accurate to develop predictive binding models for each protein, similar to the PTBP1 model here, and then to incorporate each of these binding models into a larger network model. Such an approach will allow the analysis of the many overlapping regulatory programs controlled by RNA binding proteins.

## Materials and Methods

### Hidden Markov Model for PTBP1 binding affinity prediction

A Hidden Markov Model (HMM) was designed and trained by an expectation–maximization (EM) method (Baum-Welch algorithm) using published PTBP1 CLIP data [13], [29], [30]. In total, 48,604 PTBP1-CLIP cluster sequences were used to train model parameters. During the training step, multiple initial values were tested to avoid a local maximum problem. Trained parameters included emission probabilities for nucleotide triplets, initial probabilities and transition probabilities between states [29], [30].

The trained model was used to score RNA sequences. The raw PTBP1 binding score is defined as a log-odds ratio that compares the score of a sequence from the HMM over the score from a background model. Since CLIP experiments do not have an inherent corresponding negative dataset, we generated computational negative datasets and tested different background models (Figure S4). We found that a background model that values all triplets equally yielded the most accurate binding scores [29]. Raw scores were further normalized and converted to z-scores. For the 69 mer RNA sequences used in binding assays, scores were normalized by 100,000 random sequences with same length (Figure S3). This yielded very accurate predictions of binding affinity (Figure 2).

When considering binding scores in genomic sequence, exons and upstream or downstream intron regions have different base compositions and will yield different average binding scores. Thus, to score binding sites adjacent to possible regulated exons, it is more informative to score sites relative to equivalent sequence regions. From the annotated mouse genome, we retrieved 168,111 internal exons and their flanking introns as separate sequence sets using a python library, *Pygr*. We scored log odds of these sequences with the trained model. Since the lengths and base compositions of intronic and exonic sequences are different, and binding scores automatically increase with length (Figure S13) [29], we grouped sequences by their location and sequences in each group were sorted according to length into bins of 1000 sequences each. The average score and standard deviation were determined for each bin. These values were used to transform the raw scores into z-scores for each upstream intron, downstream intron, and exon sequence. We localized the PTBP1 binding sites along each RNA sequence using the Viterbi algorithm [29], [30].

### Validation of PTBP1 binding model scores by binding assay

To test predicted PTBP1 binding scores, we selected thirteen mouse exon/intron RNA sequences (69 nucleotides) exhibiting a range scores. In the selection, other sequence features such as secondary structure were not considered. Target RNAs were transcribed *in vitro* from dsDNA using T7 RNA polymerase and subjected to an electrophoretic mobility shift assay (EMSA). During the transcription, radioactive α-32P UTP was incorporated into RNA to visualize the probes. The RNA probes were then denatured for 2 min at 85°C and cooled down on ice immediately to reduce secondary structure formation. Binding assays were carried out as previously described with some modifications [27]. Specifically, each gel mobility shift reaction (10 µL) contained the indicated amounts of recombinant human PTBP1 in 6 µL DG buffer (20 mM Hepes-KOH ph 7.9, 20% glycerol, 80 mM potassium glutamate, 0.2 mM EDTA, 0.2 mM PMSF), 1 µL 22 mM MgCl2, 1 µL 0.5 mg/ml tRNA, 0.5 µL RNase inhibitor (20 unit, RNaseOut from invitrogen), 0.5 µL DEPC treated H2O, and 1 µL 100 nM RNA probe. At first, all reaction components excluding RNase inhibitor, tRNA, and RNA probes were mixed and incubated for 8 min at 30°C. Then RNase inhibitor and tRNA were added and mixed. RNA probe was then added and the reaction was incubated for an additional 15 min. The reactions were put on ice for 5 min and mixed with 1.2 µL glycerol loading dye (30% glycerol). They were separated on 8% native polyacrylamide gels with 25 mM Tris-Gly running buffer in a cold room. Gels were dried and exposed to a phosphor screen. Then images were scanned using Typhoon 9410 and quantified using ImageQuant TL program (GE Lifesciences). The apparent *Kd* values were estimated by fitting the data to non-linear curves using Prism software.

### Logistic regression model for PTBP1 dependent exon prediction

An exon training set was compiled from previous microarray and RT-PCR experiments [20], [35]. The training set was composed with 68 PTBP1 repressed, 37 PTBP1 enhanced, and 69 non-PTBP1 regulated simple cassette exons. We only considered exons with canonical splice sites (GU-AG). An exon was classified as PTBP1 repressed or enhanced when 1) the inclusion level (PSI) of its minor isoform was greater than 5% in both the control and knock-down samples and 2) the inclusion level of its minor isoform was changed by 30% or more in the *Ptbp1* knock down condition compared to the control sample. Next, we collected sequence features for each exon and its flanking exons. The features included PTBP1 binding scores, 5′ and 3′ splice site strengths, exon/intron lengths, and word frequencies. The PTBP1 binding scores were calculated from the PTBP1 binding model described above. The strength of splice sites was calculated by the splice-site analyzer tool [37]. Using a mouse whole internal exon set, we normalized features and fed them into the model. The PTBP1 splicing model is based on a multinomial logistic regression framework using the following steps: 1) selection of initial variables with a moderate level of association (p-value from t-test<0.25), 2) removal of outlier exons, 3) stepwise variable selection [38]. We scored mouse internal exons with the trained PTBP1 splicing model and validated candidate exons with RT-PCR and RNA-seq experiments. Exons from the training set were excluded from the validation.

### Validation of exon candidates by RT-PCR and RNA-seq

To test alternative splicing events for candidate exons, we assayed exon inclusion levels in cells following *Ptbp1*, *Ptbp2*, and double *Ptbp1* & *Ptbp2* knock down. The knockdown experiment was performed as described previously with minor modification [20]. Mouse neuroblastoma (N2A) cells were cultured in DMEM with 10% FBS and 2 mM L-glutamine. At 70 to 80% confluency, cells were trypsinized and suspended in the growth medium. DNA–Lipofectamine 2k (Invitrogen) complexes were prepared and mixed with cells in a tube according to manufacturer's instructions. Tubes were incubated for 5 h with mixing every half hour. Then cells were centrifuged and cultured in plates for 3 d. Proteins and RNA was extracted from collected cells. Protein samples were subjected to fluorescence immunoblotting to monitor knockdown efficiency of *Ptbp1* and *Ptbp2*. Total RNA was collected using Trizol (Invitrogen) according to the manufacturer's instructions. The RNA was further treated with DNase I to avoid DNA contamination. For RT-PCR (Reverse Transcription-PCR) assays, the RNA was reverse transcribed to cDNA with random hexamers using SuperScript enzyme (Invitrogen) following the manufacturer's instructions. PCR reactions were performed to assay alternative splicing of particular target exons. First, forward and reverse PCR primers were designed for the flanking exons using PRIMER3 program [56]. To label PCR products, a 5′ fluorescent-labeled universal primer (5′-FAM-CGTCGCCGTCCAGCTCGACCAG-3′) was added to the PCR reaction and a universal priming site was introduced to the 5′ end of the forward primer (5′-CGTCGCCGTCCAGCTCGACCAG-Forward Primer-3′). Each PCR reaction (15 µL) was carried out with 1.5 picomole of the forward primer and 6.75 picomole of the reverse and universal primers [57]. PCR amplification proceeded with an initial denaturation at 94°C for 4 m followed by 24 cycles of 94°C for 30 s, at a melting temperature of the reverse primer for 45 s, and 72°C for 45 s, with a final extension step at 72°C for 10 m. The samples were mixed with 2× formamide buffer (Formamide with 1 mM EDTA pH 8.0) and denatured at 95°C for 5 min. Then samples were chilled on ice and run on 8% denaturing polyacrylamide gels. Gels were directly scanned by Typhoon and quantified by ImageQuant program.

RNA-seq libraries were constructed following standard protocols (Illumina TruSeq RNA Sample Prep Kit). To make strand-specific libraries, we added two extra steps to the protocol [41]. After first strand cDNA synthesis, remaining dNTPs were removed by a size selection on beads (AMPure XP). Second-strand cDNA was synthesized with a dNTP mix containing dUTP instead of dTTP. The reaction contained samples eluted in 50 µl resuspension buffer, 2 µl 5× FS buffer, 1 µl 50 mM MgCl2, 1 µl 100 mM DTT, 2 µl 10 mM dUTP nucleotides mix, 15 µl Second Strand Buffer (Invitrogen), 0.5 µl E.coli DNA Ligase (10 U/µl;NEB), 0.5 µl RNase H (2 U/µl;Invitrogen), 2 µl DNA E.coli Polymerase I (10 U/µl;NEB). The reaction was incubated for 2 h at 16°C. After sequencing adaptors were ligated, 1 µl USER (Uracil-Specific Excision Reagent enzyme; NEB) was added to reactions to degrade the second strand cDNA. The samples were incubated for 15 min at 37°C and the reaction were inactivated at 94°C for 5 min. The samples were put in ice and then subjected to PCR amplification. Average size of inserts was about 225 bp and the libraries were subjected to 100 bp paired-end sequencing (Illumina HiSeq2000 platform). Using SpliceTrap [42], 60–65% of reads were mapped to exon duos or trios. In total, 180M (179,511,116) and 145M (145,334,711) paired end reads were used to infer exon inclusion ratios in the control and *Ptbp1* knockdown conditions, respectively. The data have been deposited in NCBI's Gene Expression Omnibus [58] and are accessible through GEO Series accession number GSE45119.

## Supporting Information

Figure S1 **Five-fold cross-validation of the PTBP1 binding model using Hela CLIP clusters.** To test the two state model of binding and non-binding triplets, we divided the CLIP-data to the five subsets. In each plot, four sub sets were used in training the model and one subset was subjected to scoring. We then compared scores from the CLIP-subset sequences to random sequences picked from same genic regions that contained the CLIP clusters. As shown, sequences from CLIP-subset generated significantly higher scores than random. The results indicate that the triplets identified by the HMM as predictive of state 1 are predictive of PTBP1 CLIP sites and thus of protein binding.
(TIF)

Figure S2 The density of PTBP1 binding triplets predicted by the HMM peaks at the aligned crosslink sites from Human ESC iCLIP clusters.
(TIF)

Figure S3 **PTBP1 binding model scores and validation.** **A**. Summary statistics and the distribution of raw and normalized PTBP1 binding scores for 100,000 random sequences. **B**. Electrophoretic mobility shift assay of RNAs with various PTBP1 binding scores. RNAs were transcribed in vitro, incubated with increasing concentrations of purified PTBP1 (0 to 200 nM), and the bound and unbound RNA separated on native gels. Arrows indicate RNA-protein complexes. The fraction of PTBP1-bound RNA is plotted below for each RNA.
(TIF)

Figure S4 **Evaluation of different background models for scoring PTBP1 binding.** Four background models were evaluated. The uniform distribution model assumes equal frequencies of triplets. The PTBP1 target gene set model used random sequences from genes containing PTBP1 CLIP clusters. The two shuffled models used shuffled CLIP cluster sequences maintaining mono or di nucleotide ratios. We calculated PTBP1 binding scores based on each background model and compared the scores to the measured dissociation constants. Based on the rank correlation, the uniform distribution model worked best. The PTBP1 target gene set model showed comparable performance. It slightly improves the linear fit (−0.91 vs. −0.95) for some strong binders. However, it wrongly predicted some binders as non-binders, which reduced the rank correlation (−0.95 to −0.90). The two shuffled models did not perform well.
(TIF)

Figure S5 **Correlations of particular sequence features with PTBP1 repression.** For PTBP1-repressed and PTBP1 non-regulated exons, we calculated scores for sequence features and determined the fraction of PTBP1-repressed exons in each score bin. Shown are the graphs for the 3′ splice site score, and the PTBP1 binding scores in the upstream intron, the exon, and the downstream intron plotted against the percent of exons within the score bin that are PTBP1-repressed.
(TIF)

Figure S6 **Performance of PTBP1-dependent splicing models.** **A**. Receiver Operating Characteristic Curves in a leave-one-out cross validation for each logit: exon repression (*left*) and exon enhancement (*right*). **B**. Sensitivity and specificity plotted across the whole threshold range. Sensitivity is defined as the percent of true repressed exons that are correctly predicted as repressed at the corresponding threshold. Specificity is defined as the percent of actual non-repressed exons that are correctly predicted as non-repressed at the corresponding threshold.
(TIF)

Figure S7 **ShRNA mediated depletion of PTBP1 and PTBP2.** Duplicate immunoblots after shRNA knockdown of PTBP1, PTBP2 or both proteins. Note that depletion of PTBP1 induces expression of PTBP2 as observed previously. Numbers above each lane indicate the fluorescence intensity for PTBP1 or PTBP2 relative to the control lane.
(TIF)

Figure S8 **Characteristics of false positive exons.** **A**. Exon inclusion was measured for three false positives exons after *Ptbp1* knockdown (left), or *Ptbp2* knockdown, and *Ptbp1* & *Ptbp2* double knockdown (right). P-values were calculated from biological triplicates using paired one-tailed t-tests. **B**. False positive exons exhibit higher PSI values prior to PTBP1 depletion. Box plot of exon inclusion for twenty-nine false positive exons showing little change in splicing by RNA-seq after PTBP1 depletion (delta PSI<5%) that score with high probability to be repressed (>0.55). Exon inclusion levels prior to PTBP1 depletion are compared to thirty-six true positive exons.
(TIF)

Figure S9 **Two exons with intermediate scores for PTBP1 repression show complex responses to PTBP1 and PTBP2 depletion.** Exon inclusion was measured after *Ptbp1* depletion (left), or after *Ptbp2* and *Ptbp1/Ptbp2* double depletion (right). P-values were calculated from biological triplicates using paired one-tailed t-tests.
(TIF)

Figure S10 **PTBP2 dependence of predicted PTBP1 target exons.** RT-PCR of high probability PTBP1 exon targets following *Ptbp2* knockdown or *Ptbp2/Ptbp1* double knockdown. Relative band intensities of the gels in triplicate on the right are plotted on the left to show the average delta PSI ± SE (Percent Spliced In). P-values were calculated from paired one-tailed t-tests with PSI values in control samples.
(TIF)

Figure S11 **PTBP2 dependence of predicted non-PTBP1 target exons.** RT-PCR of exon with low probabilities for PTBP1 repression (≤0.2). *Ptbp2* knockdown and *Ptbp1/Ptbp2* double knock down with data analysis as in Figure S10.
(TIF)

Figure S12 **Boxplot of PSI (Percent of Spliced In) values estimated from splicing sensitive microarray data for exons expressed in mouse C2C12 myoblasts.** Exons with higher (>0.65) and lower (<0.2) repression probabilities are compared. Exons used in the original training set were excluded from the plot. The number of exons in the corresponding probability bin is given by *n*. The p-value was calculated from a one-tailed Student's t-test.
(TIF)

Figure S13 **Distribution of PTBP1 binding scores of exons and introns before and after normalization.** 168,111 exons and their flanking introns from the set of annotated mouse internal exons were subjected to scoring and normalization. Raw PTBP1 Binding scores are affected by sequence length and base composition. To account differences in these features between introns and exons in normalized scores, we grouped the exons and their upstream and downstream introns separately. The sequences in each group were sorted according to length into bins of 1,000 sequences each. The average scores and standard deviations were determined for each bin. These values were used to transform the raw scores into z-scores for each sequence per bin.
(TIF)

Table S1 **Trained PTBP1 splicing regulation model.** The table presents a summary of the multinomial logistic regression model for PTBP1 splicing regulation, including estimated coefficients and their statistics.
(TIF)

Table S2 **Enriched gene ontology categories for novel PTBP1-repressed exons.** The table lists ontology entries enriched in genes with predicted PTBP1-repressed exons (probability score of exon repression >0.65). Whole mouse internal exons were used as the control set, and p-value cut off was 0.05. Gene ontology analysis was performed using the GOTM web server.
(TIF)
