# Supplementary figures and images for: De Novo Prediction of PTBP1 Binding and Splicing Targets Reveals Unexpected Features of Its RNA Recognition and Function
Source: PLoS Comput Biol. 2014 Jan 30;10(1):e1003442. doi: 10.1371/journal.pcbi.1003442 (PMC3907290; doi:10.1371/journal.pcbi.1003442)

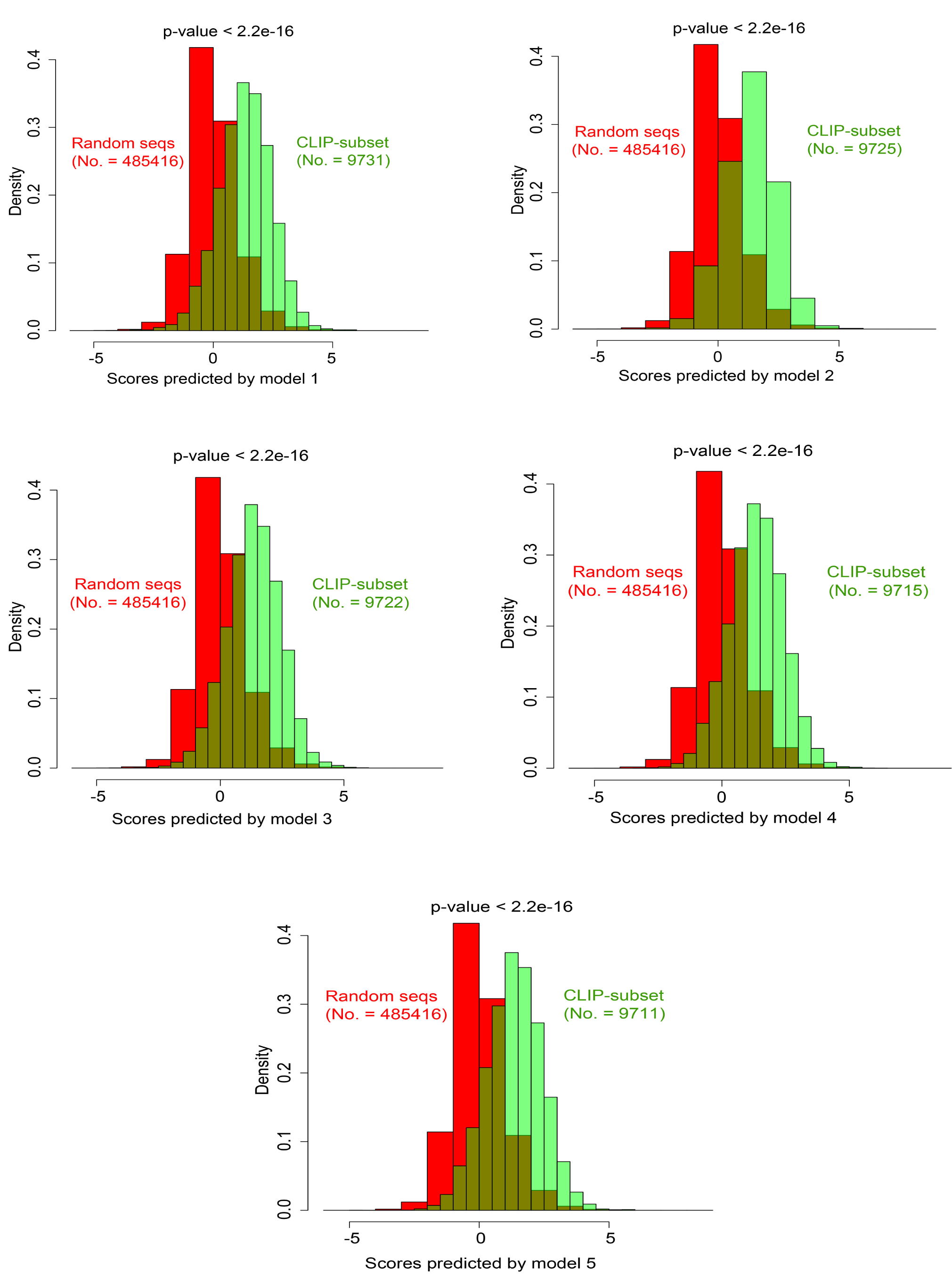

Supplement: Figure S1 — Five-fold cross-validation of the PTBP1 binding model using Hela CLIP clusters. To test the two state model of binding and non-binding triplets, we divided the CLIP-data to the five subsets. In each plot, four sub sets were used in training the model and one subset was subjected to scoring. We then compared scores from the CLIP-subset sequences to random sequences picked from same genic regions that contained the CLIP clusters. As shown, sequences from CLIP-subset generated significantly higher scores than random. The results indicate that the triplets identified by the HMM as predictive of state 1 are predictive of PTBP1 CLIP sites and thus of protein binding. (TIF) [file pcbi.1003442.s001.tif]

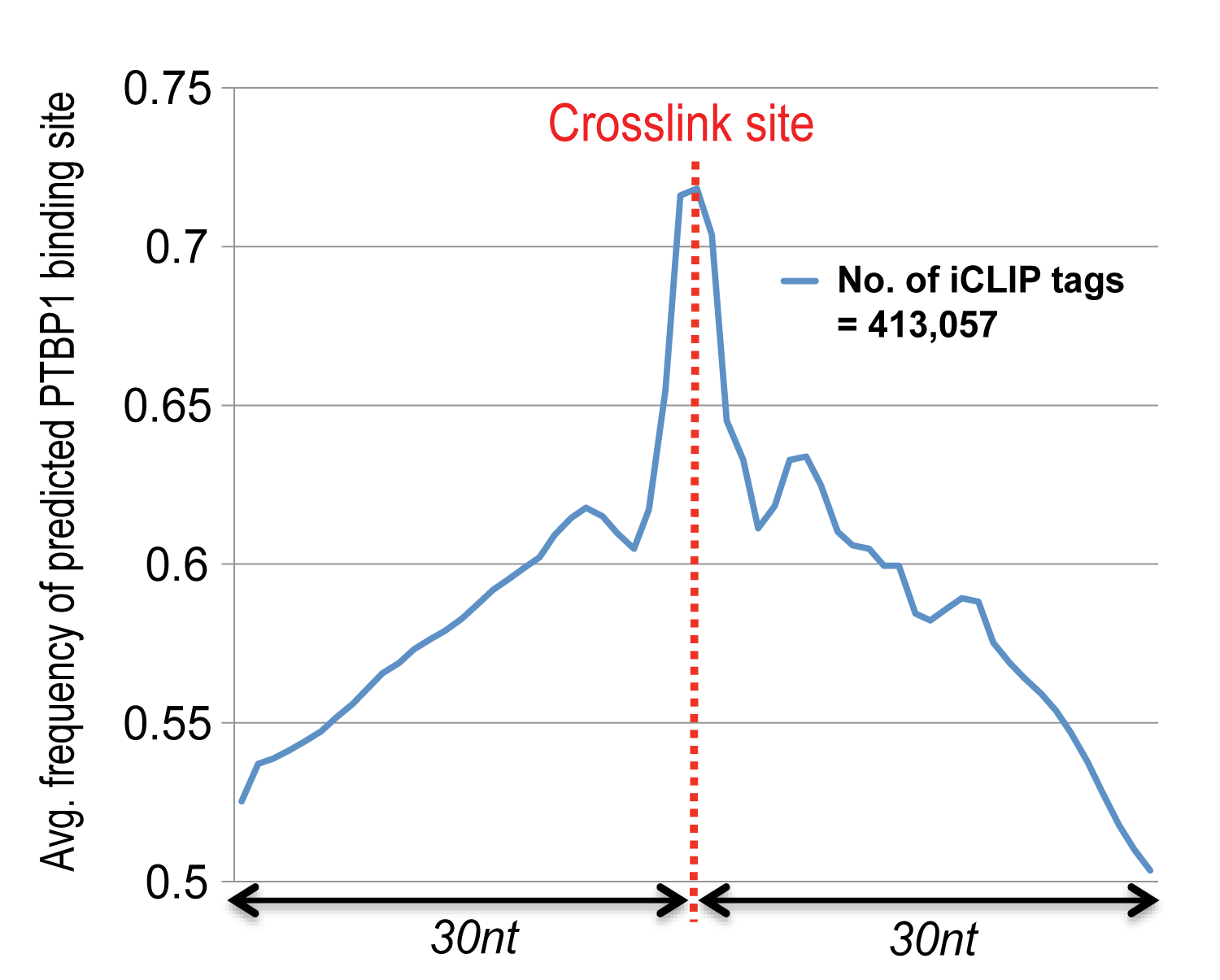

Supplement: Figure S2 — The density of PTBP1 binding triplets predicted by the HMM peaks at the aligned crosslink sites from Human ESC iCLIP clusters. (TIF) [file pcbi.1003442.s002.tif]

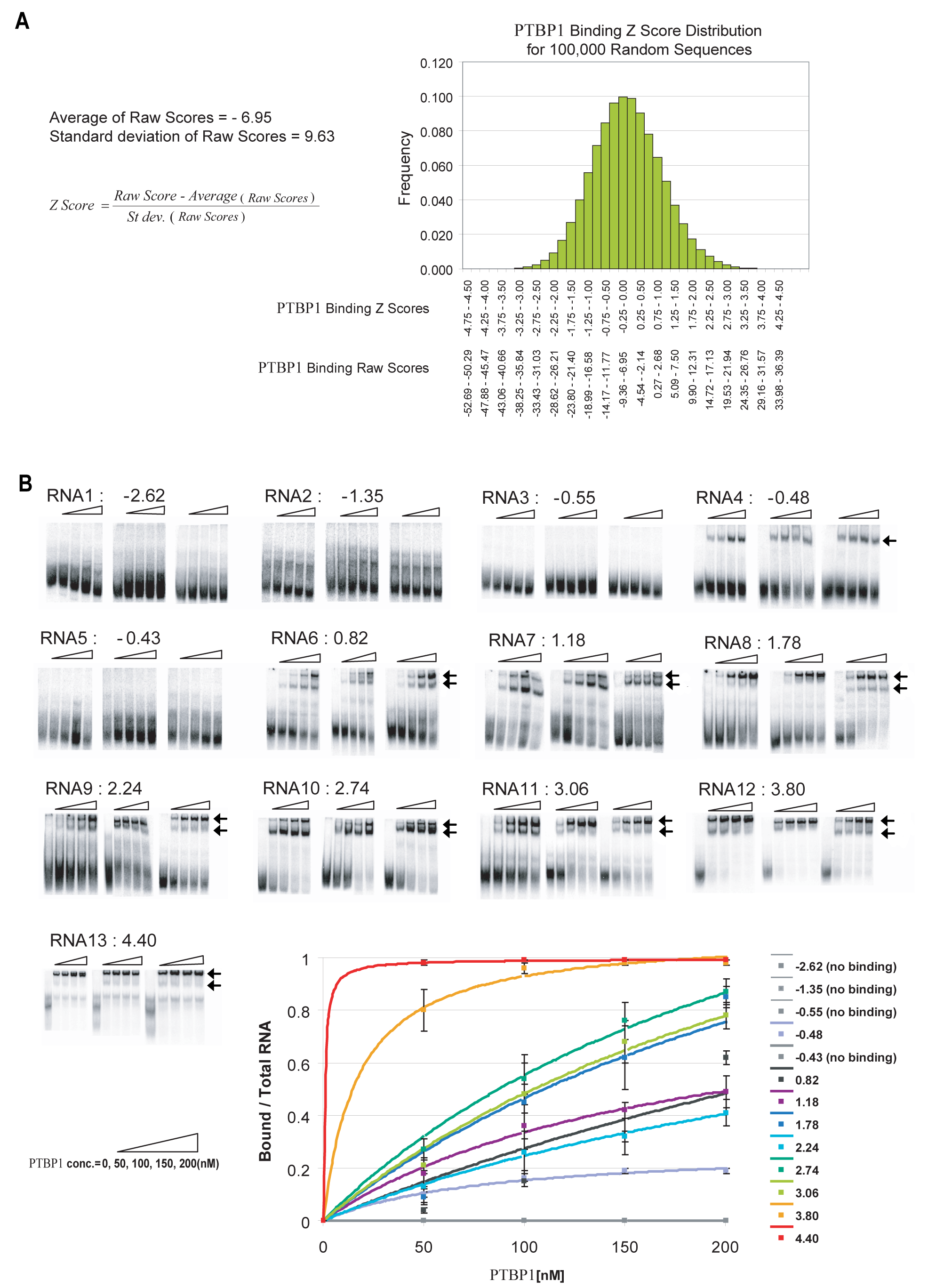

Supplement: Figure S3 — PTBP1 binding model scores and validation. A. Summary statistics and the distribution of raw and normalized PTBP1 binding scores for 100,000 random sequences. B. Electrophoretic mobility shift assay of RNAs with various PTBP1 binding scores. RNAs were transcribed in vitro, incubated with increasing concentrations of purified PTBP1 (0 to 200 nM), and the bound and unbound RNA separated on native gels. Arrows indicate RNA-protein complexes. The fraction of PTBP1-bound RNA is plotted below for each RNA. (TIF) [file pcbi.1003442.s003.tif]

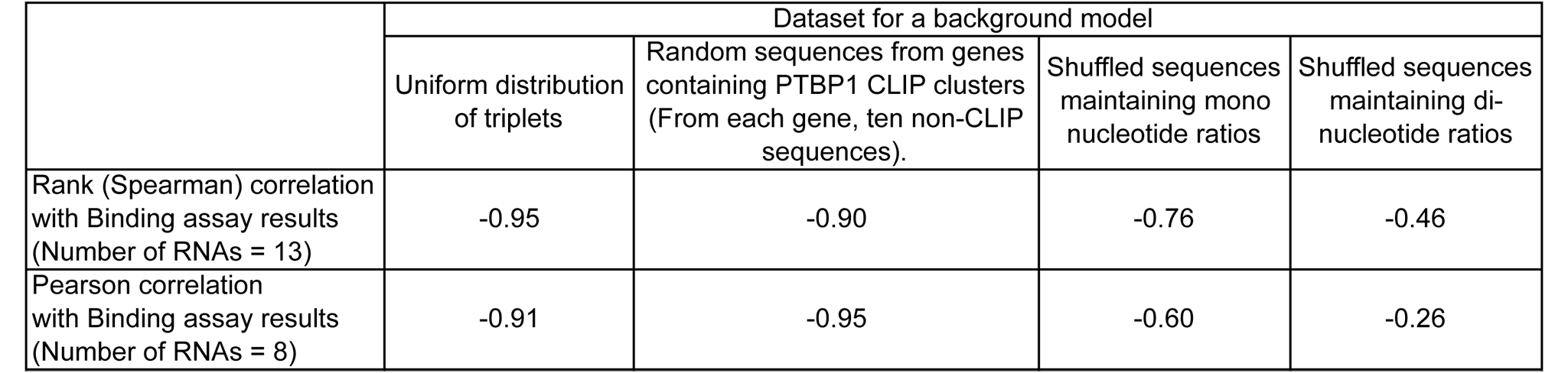

Supplement: Figure S4 — Evaluation of different background models for scoring PTBP1 binding. Four background models were evaluated. The uniform distribution model assumes equal frequencies of triplets. The PTBP1 target gene set model used random sequences from genes containing PTBP1 CLIP clusters. The two shuffled models used shuffled CLIP cluster sequences maintaining mono or di nucleotide ratios. We calculated PTBP1 binding scores based on each background model and compared the scores to the measured dissociation constants. Based on the rank correlation, the uniform distribution model worked best. The PTBP1 target gene set model showed comparable performance. It slightly improves the linear fit (−0.91 vs. −0.95) for some strong binders. However, it wrongly predicted some binders as non-binders, which reduced the rank correlation (−0.95 to −0.90). The two shuffled models did not perform well. (TIF) [file pcbi.1003442.s004.tif]

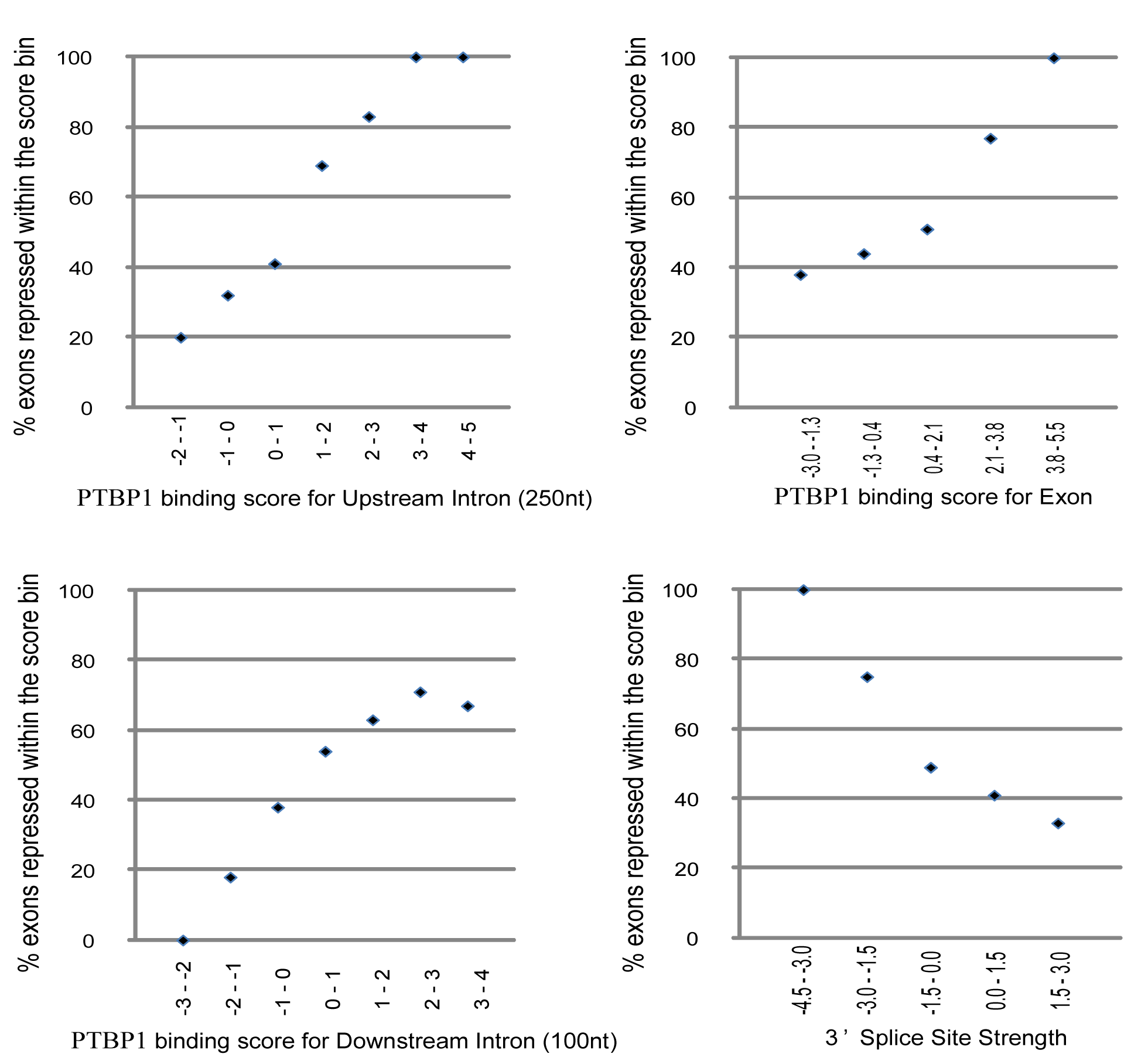

Supplement: Figure S5 — Correlations of particular sequence features with PTBP1 repression. For PTBP1-repressed and PTBP1 non-regulated exons, we calculated scores for sequence features and determined the fraction of PTBP1-repressed exons in each score bin. Shown are the graphs for the 3′ splice site score, and the PTBP1 binding scores in the upstream intron, the exon, and the downstream intron plotted against the percent of exons within the score bin that are PTBP1-repressed. (TIF) [file pcbi.1003442.s005.tif]

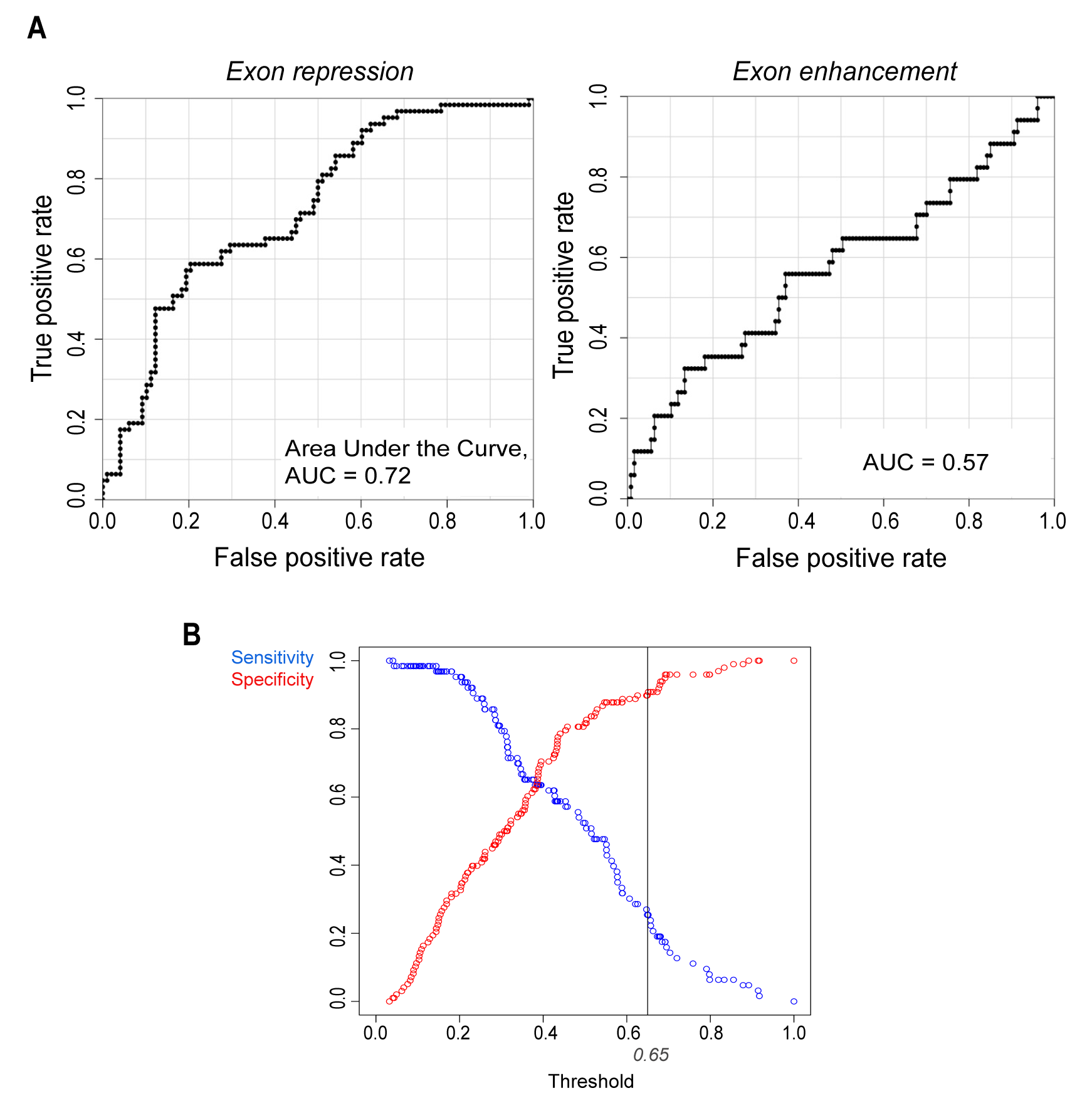

Supplement: Figure S6 — Performance of PTBP1-dependent splicing models. A. Receiver Operating Characteristic Curves in a leave-one-out cross validation for each logit: exon repression (left) and exon enhancement (right). B. Sensitivity and specificity plotted across the whole threshold range. Sensitivity is defined as the percent of true repressed exons that are correctly predicted as repressed at the corresponding threshold. Specificity is defined as the percent of actual non-repressed exons that are correctly predicted as non-repressed at the corresponding threshold. (TIF) [file pcbi.1003442.s006.tif]

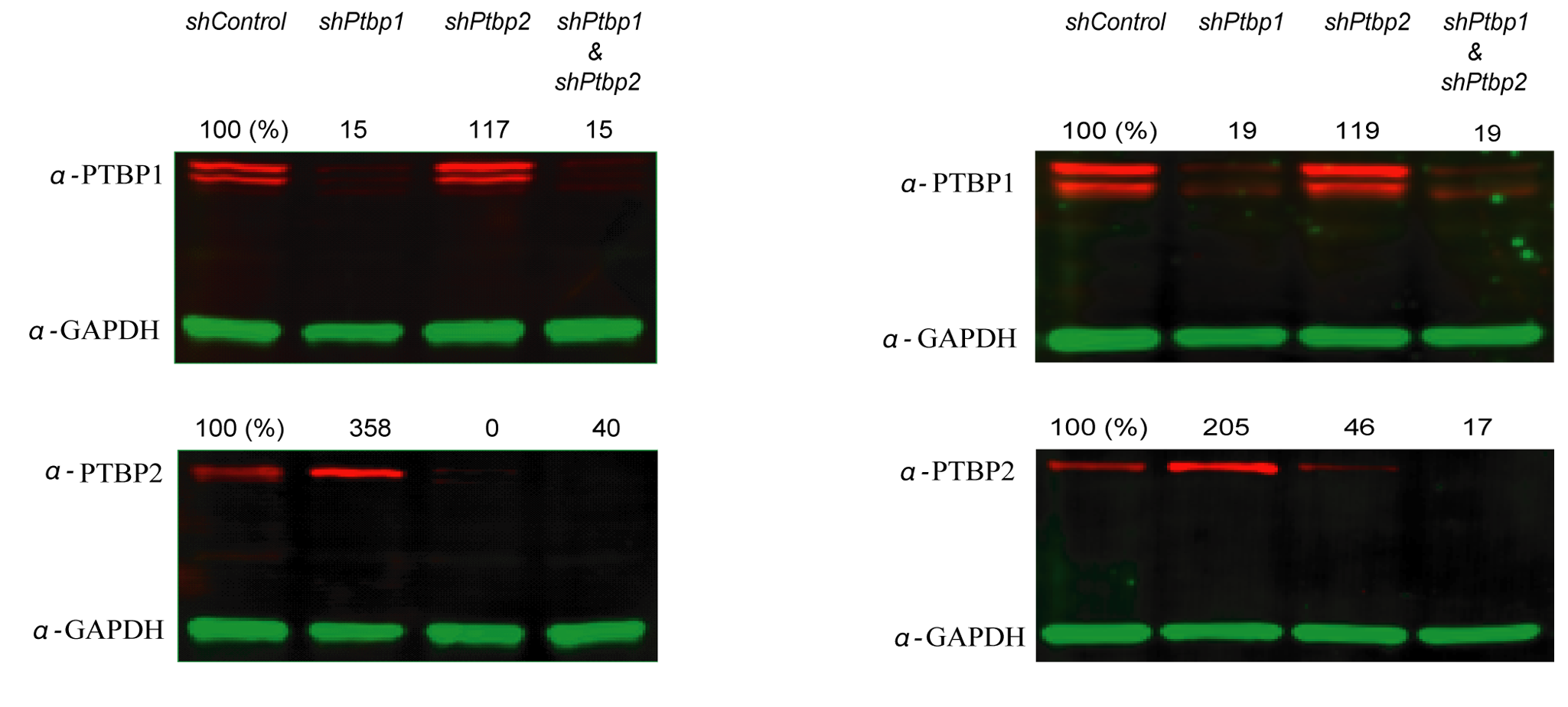

Supplement: Figure S7 — ShRNA mediated depletion of PTBP1 and PTBP2. Duplicate immunoblots after shRNA knockdown of PTBP1, PTBP2 or both proteins. Note that depletion of PTBP1 induces expression of PTBP2 as observed previously. Numbers above each lane indicate the fluorescence intensity for PTBP1 or PTBP2 relative to the control lane. (TIF) [file pcbi.1003442.s007.tif]

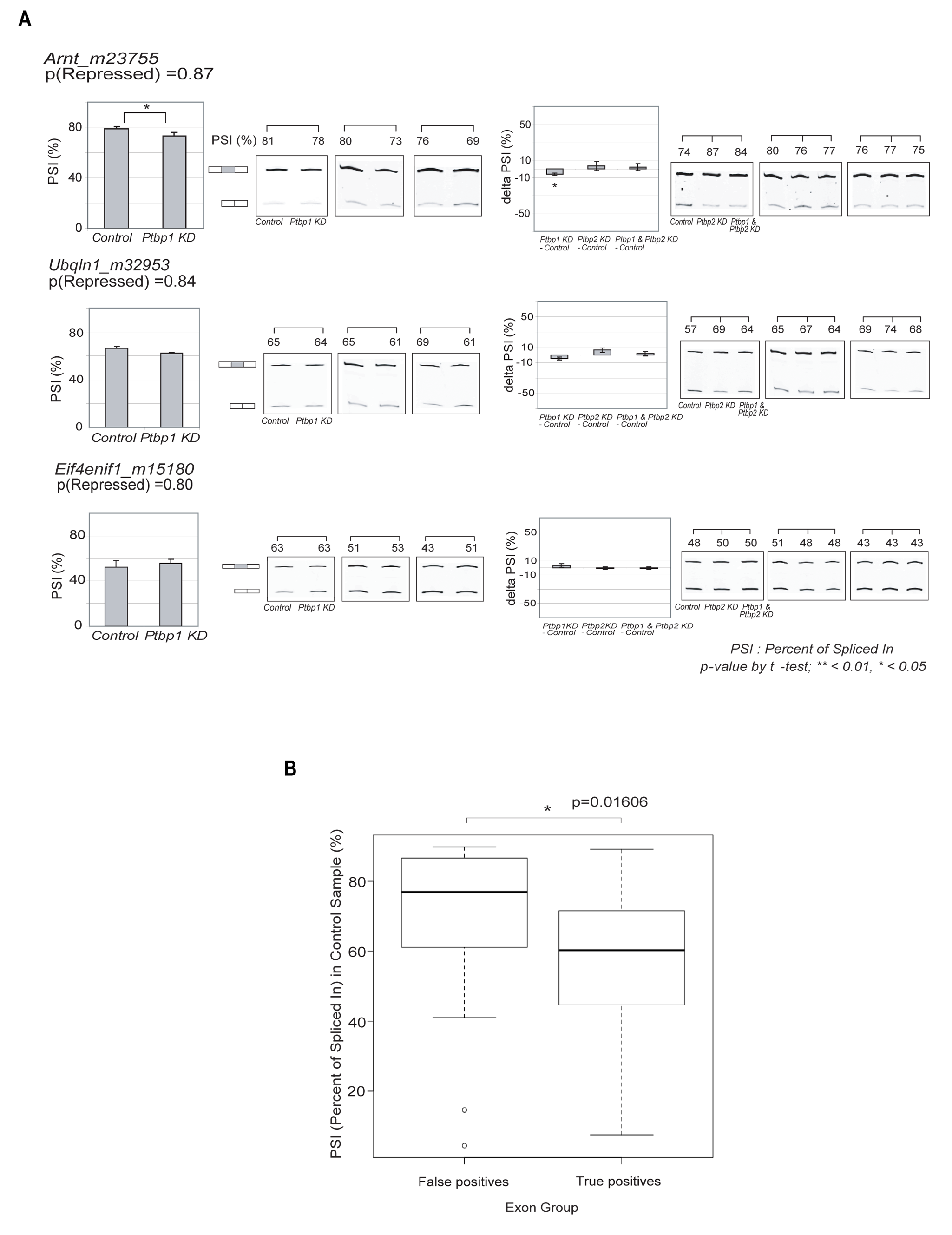

Supplement: Figure S8 — Characteristics of false positive exons. A. Exon inclusion was measured for three false positives exons after Ptbp1 knockdown (left), or Ptbp2 knockdown, and Ptbp1 & Ptbp2 double knockdown (right). P-values were calculated from biological triplicates using paired one-tailed t-tests. B. False positive exons exhibit higher PSI values prior to PTBP1 depletion. Box plot of exon inclusion for twenty-nine false positive exons showing little change in splicing by RNA-seq after PTBP1 depletion (delta PSI<5%) that score with high probability to be repressed (>0.55). Exon inclusion levels prior to PTBP1 depletion are compared to thirty-six true positive exons. (TIF) [file pcbi.1003442.s008.tif]

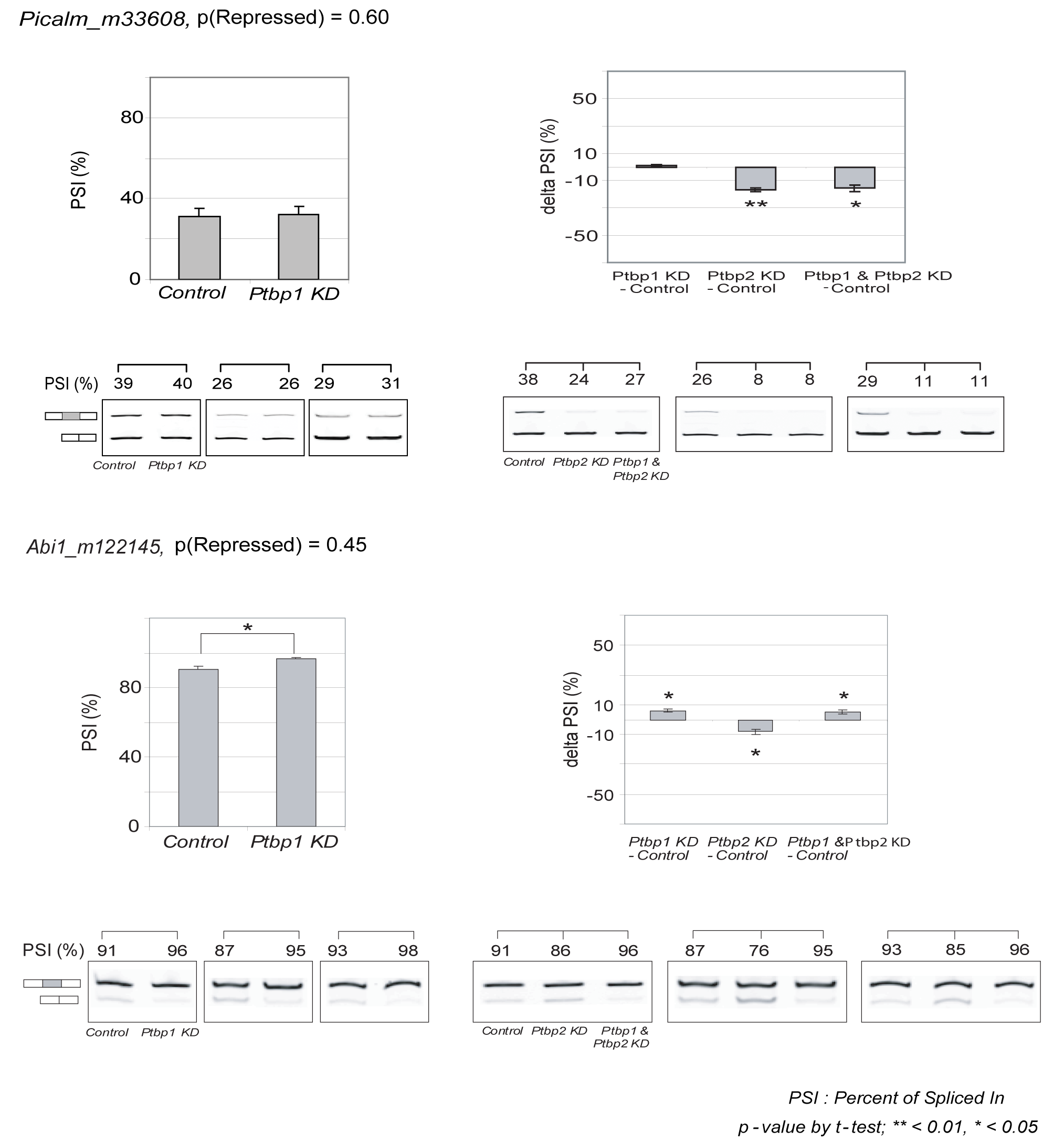

Supplement: Figure S9 — Two exons with intermediate scores for PTBP1 repression show complex responses to PTBP1 and PTBP2 depletion. Exon inclusion was measured after Ptbp1 depletion (left), or after Ptbp2 and Ptbp1/Ptbp2 double depletion (right). P-values were calculated from biological triplicates using paired one-tailed t-tests. (TIF) [file pcbi.1003442.s009.tif]

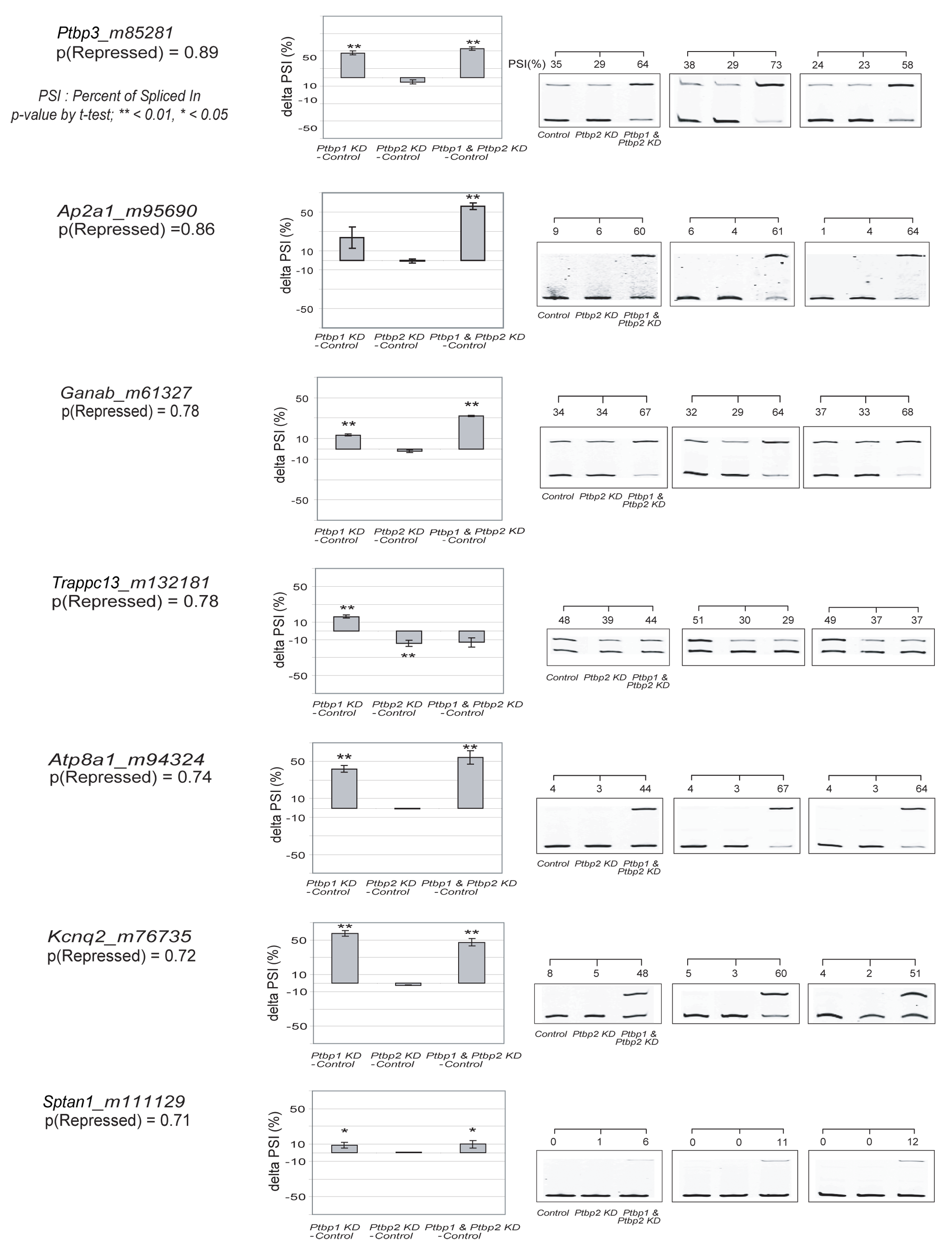

Supplement: Figure S10 — PTBP2 dependence of predicted PTBP1 target exons. RT-PCR of high probability PTBP1 exon targets following Ptbp2 knockdown or Ptbp2/Ptbp1 double knockdown. Relative band intensities of the gels in triplicate on the right are plotted on the left to show the average delta PSI ± SE (Percent Spliced In). P-values were calculated from paired one-tailed t-tests with PSI values in control samples. (TIF) [file pcbi.1003442.s010.tif]

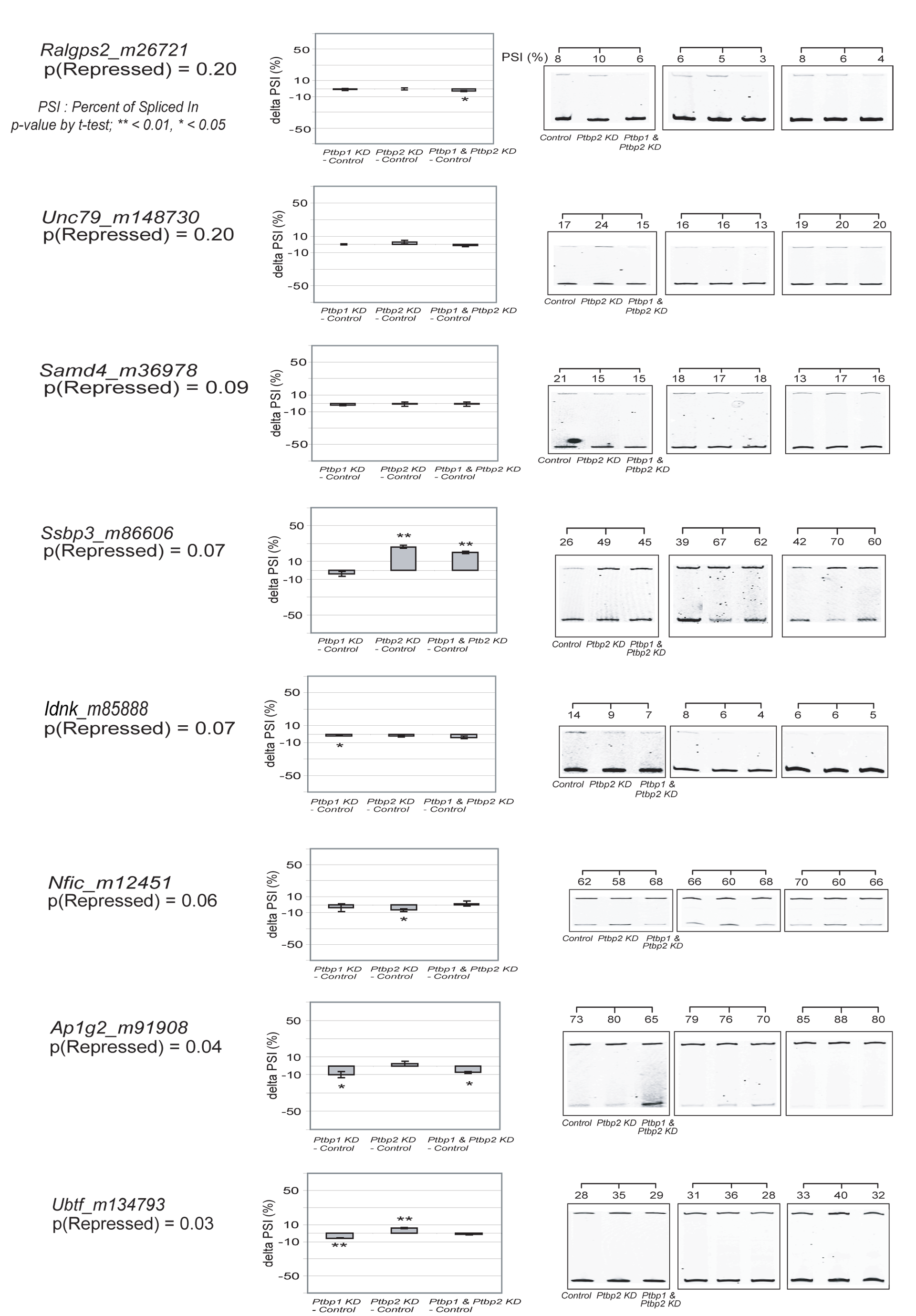

Supplement: Figure S11 — PTBP2 dependence of predicted non-PTBP1 target exons. RT-PCR of exon with low probabilities for PTBP1 repression (≤0.2). Ptbp2 knockdown and Ptbp1/Ptbp2 double knock down with data analysis as in Figure S10. (TIF) [file pcbi.1003442.s011.tif]

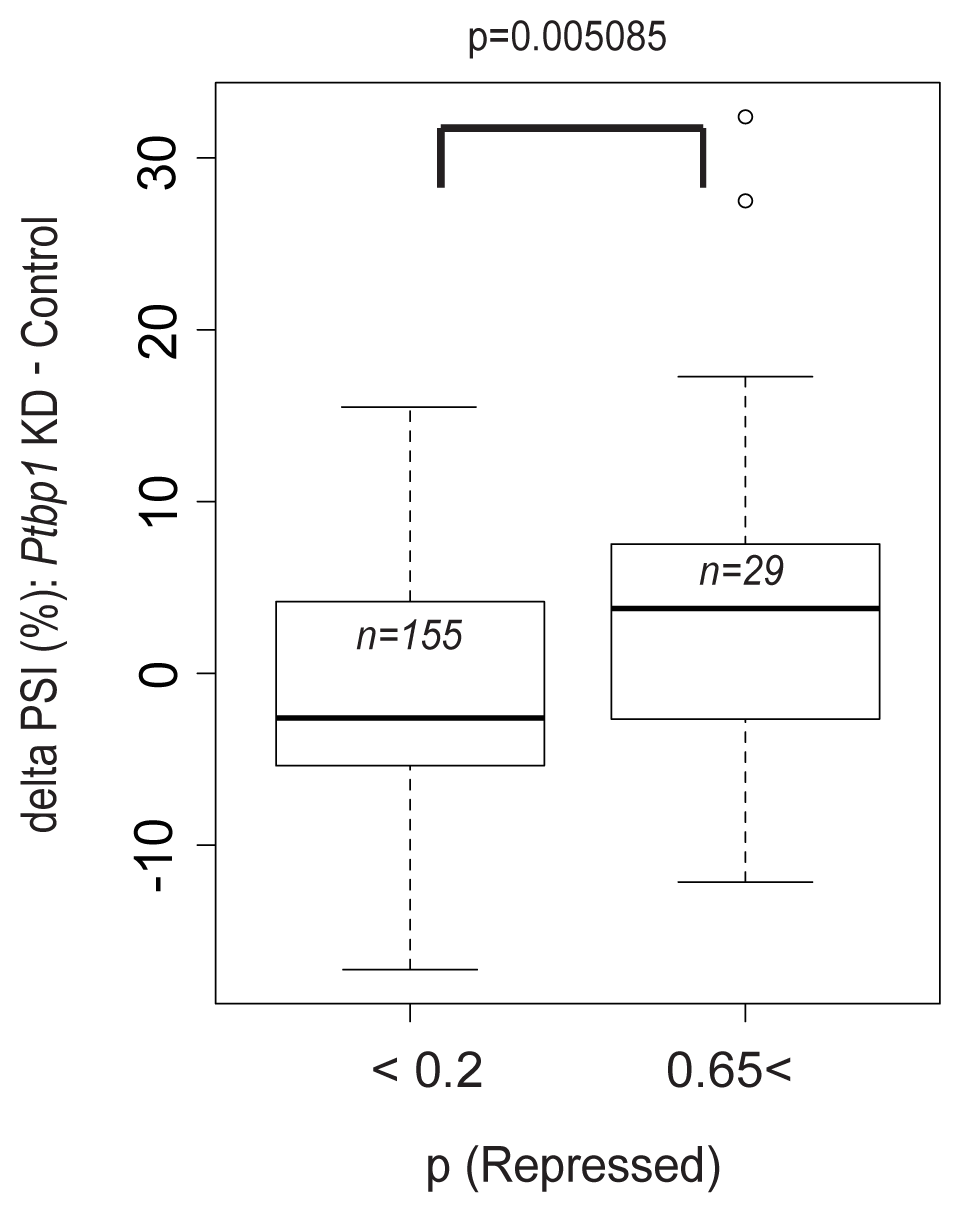

Supplement: Figure S12 — Boxplot of PSI (Percent of Spliced In) values estimated from splicing sensitive microarray data for exons expressed in mouse C2C12 myoblasts. Exons with higher (>0.65) and lower (<0.2) repression probabilities are compared. Exons used in the original training set were excluded from the plot. The number of exons in the corresponding probability bin is given by n. The p-value was calculated from a one-tailed Student's t-test. (TIF) [file pcbi.1003442.s012.tif]

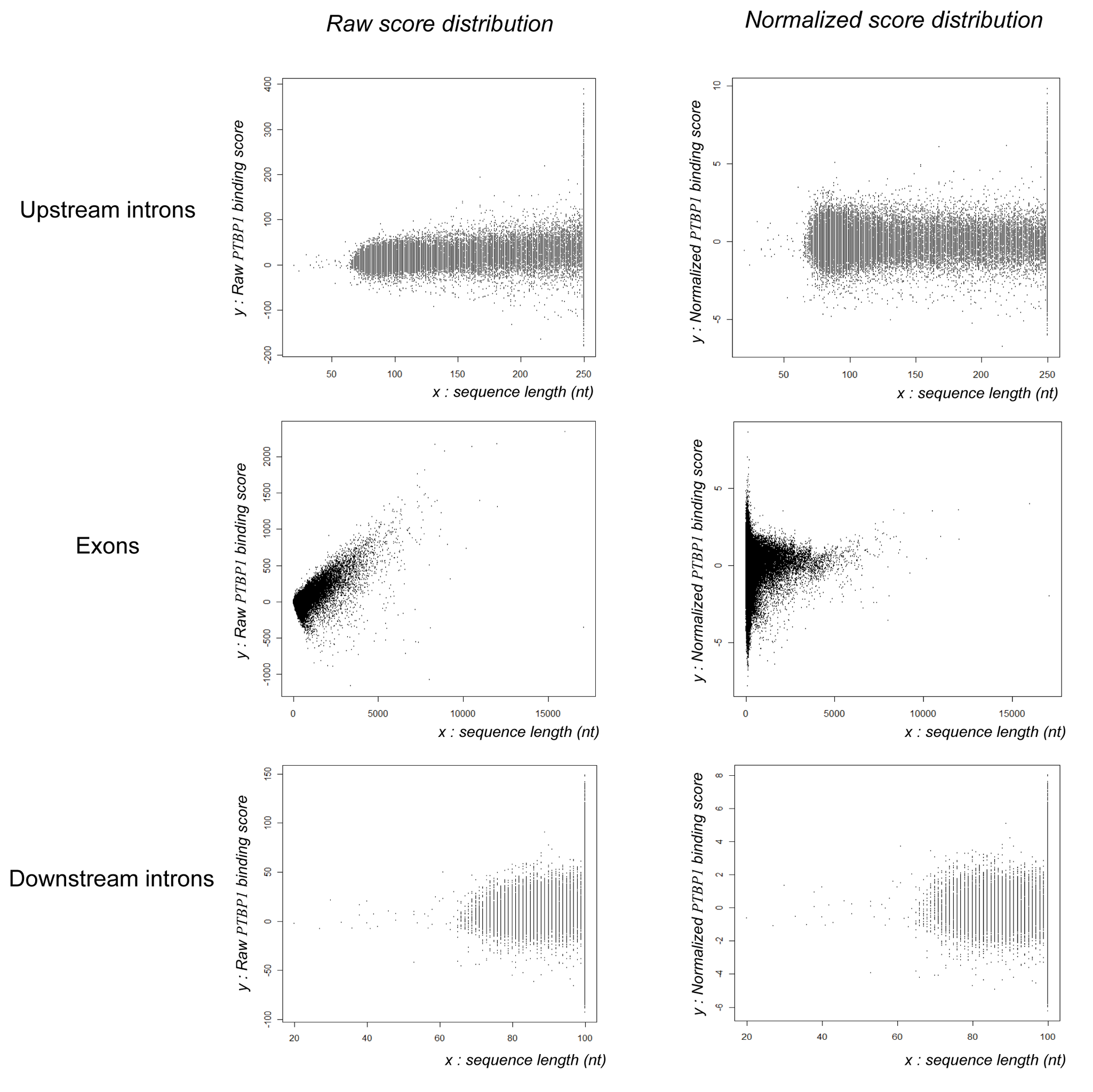

Supplement: Figure S13 — Distribution of PTBP1 binding scores of exons and introns before and after normalization. 168,111 exons and their flanking introns from the set of annotated mouse internal exons were subjected to scoring and normalization. Raw PTBP1 Binding scores are affected by sequence length and base composition. To account differences in these features between introns and exons in normalized scores, we grouped the exons and their upstream and downstream introns separately. The sequences in each group were sorted according to length into bins of 1,000 sequences each. The average scores and standard deviations were determined for each bin. These values were used to transform the raw scores into z-scores for each sequence per bin. (TIF) [file pcbi.1003442.s013.tif]

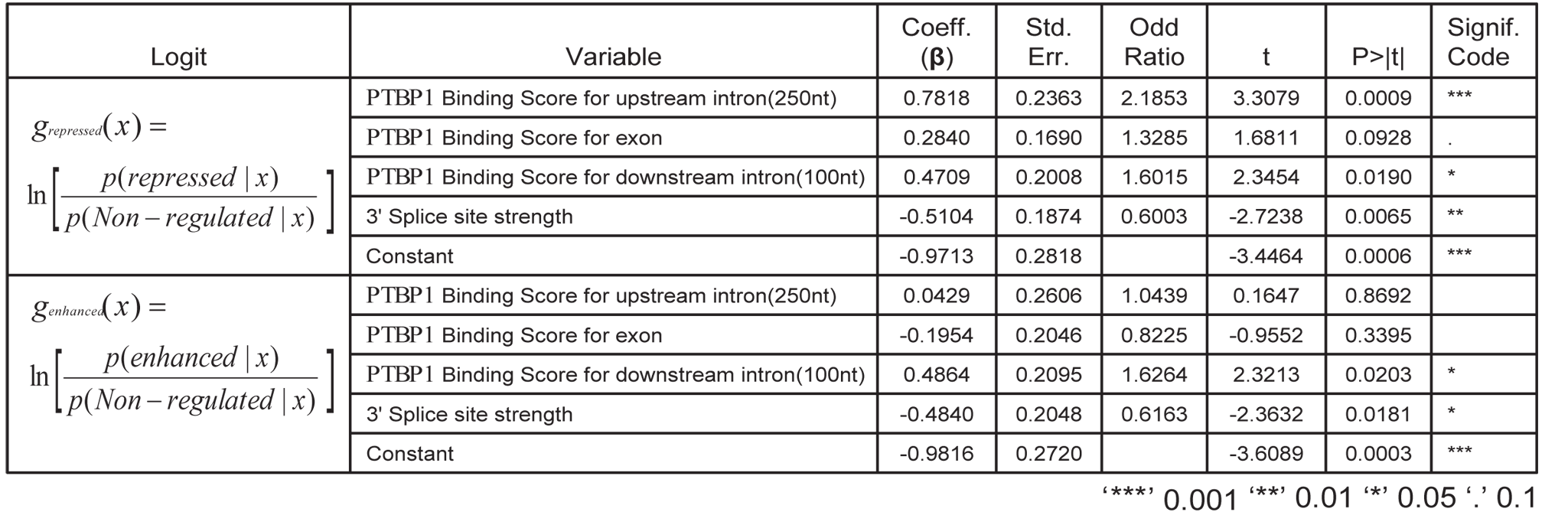

Supplement: Table S1 — Trained PTBP1 splicing regulation model. The table presents a summary of the multinomial logistic regression model for PTBP1 splicing regulation, including estimated coefficients and their statistics. (TIF) [file pcbi.1003442.s014.tif]

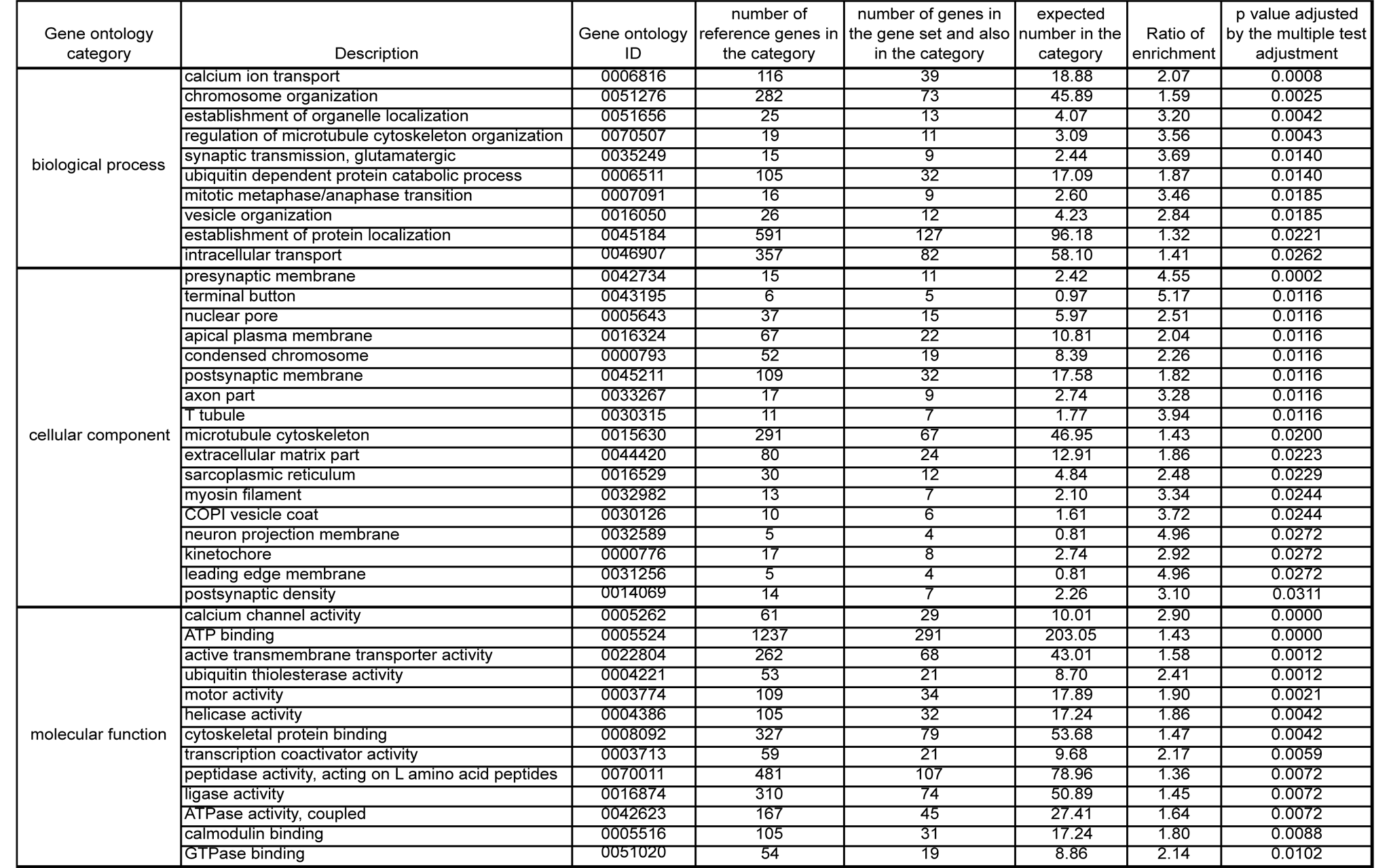

Supplement: Table S2 — Enriched gene ontology categories for novel PTBP1-repressed exons. The table lists ontology entries enriched in genes with predicted PTBP1-repressed exons (probability score of exon repression >0.65). Whole mouse internal exons were used as the control set, and p-value cut off was 0.05. Gene ontology analysis was performed using the GOTM web server. (TIF) [file pcbi.1003442.s015.tif]
